# Bimetallic Zr,Zr-Hydride Complexes in Zirconocene Catalyzed Alkene Dimerization

**DOI:** 10.3390/molecules25092216

**Published:** 2020-05-08

**Authors:** Lyudmila V. Parfenova, Pavel V. Kovyazin, Almira Kh. Bikmeeva

**Affiliations:** Institute of Petrochemistry and Catalysis of Russian Academy of Sciences, 141, Prospekt Oktyabrya, 450075 Ufa, Russia; kpv38@mail.ru (P.V.K.); almira.bikmeeva@gmail.com (A.K.B.)

**Keywords:** zirconocene, metal hydrides, methylaluminoxane, alkene dimerization, nuclear magnetic resonance

## Abstract

Being valuable precursors in the production of adhesives, lubricants, and other high-performance synthetic compounds, alkene dimers and oligomers can be obtained using homogeneous zirconocene catalytic systems. Further advances in such systems require precise control of their activity and chemoselectivity, increasing both the purity and yield of the products. This relies on the process mechanism usually built around the consideration of the hydride complexes as active intermediates in the alkene di- and oligomerization; however, the majority of studies lack the direct evidence of their involvement. Parallel studies on a well-known Cp_2_ZrCl_2_-AlR_3_ or HAlBu^i^_2_ and a novel [Cp_2_ZrH_2_]_2_-ClAlR_2_ (R = Me, Et, Bu^i^) systems activated by methylaluminoxane (MMAO-12) have shown a deep similarity both in the catalytic performance and intermediate composition. As a result of the NMR studies, among all the intermediates considered, we proved that new Zr,Zr- hydride complexes having the type x[Cp_2_ZrH_2_∙Cp_2_ZrHCl∙ClAlR_2_]∙yMAO appear to be specifically responsible for the alkene dimerization with high yield.

## 1. Introduction

**Scheme 1:**
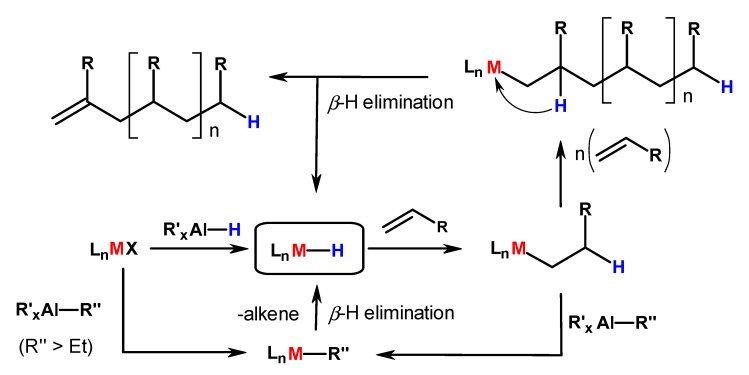
Metal hydride intermediates as active centers of alkene oligo- and polymerization [5,10,11,36].

Hydride metal complexes attract significant attention in the field of organometallic chemistry due to their ability to function as highly active reagents or catalytically active centers for various reactions. According to numerous studies, Ti subgroup complexes that contain M-H bond supposedly act as active species, for example, in di-, oligo-, and polymerization of alkenes [1,2,3,4,5], as well as in the reduction of unsaturated compounds [6,7,8]. With respect to the alkene di-, oligo-, and polymerization reactions catalyzed by both Ti subgroup metallocenes and methylaluminoxane (MAO), this assumption had been repeatedly proposed (Scheme 1); however, these studies lack direct evidence of the metal hydride complex action. Nevertheless, we note several indirect observations supporting this assumption:

(i) The presence of a terminal vinylidene group in the alkene di-, oligo- and polymerization products that appear due to the chain termination on *β*-H elimination stage which generates in situ intermediates having the M–H bond (Scheme 1) [9,10,11,12,13,14,15];

(ii) running the catalytic system in the presence of hydrogen activates “dormant” or deactivated catalytic sites and results in the acceleration of the polymerization reactions and improvement of the polymer properties [16,17,18,19];

(iii) introduction of AlBu^i^_3_ into the catalytic systems zirconocene–(methylaluminoxane or boron activators) makes such systems more effective in alkene di-, oligo-, or polymerization [19,20,21,22,23,24,25,26,27,28]; 

(iv) formation of bimetallic hydride complexes in the reactions of L_2_ZrCl_2_ with XAlBu^i^_2_ (X = H, Cl, Bu^i^) [29,30,31,32,33] which catalyze the alkene hydroalumination [29,30,31,32] and polymerization when the complexes are being transformed into the cationic species by [Ph_3_C][B(C_6_F_5_)_4_)] [34,35]. 

Moreover, Marks et al. showed that the zirconium hydride complexes modified with B(C_6_F_5_)_3_ are active in ethylene and propylene polymerization [37,38]. Collins et al. demonstrated significantly higher activity of the complexes (RCp)_2_ZrH_3_AlH_2_ (R = Bu^n^, TMS) activated with MAO in ethylene polymerization than the corresponding zirconocene dichlorides [39]. Binuclear hydride clusters complexed with organoboron compounds [40,41,42] were found to be highly active initiators of isobutene homopolymerization and isobutene-isoprene copolymerization [42]. It is generally accepted that the activating effect of MAO or organoboron compounds on di-, oligo- and polymerization systems is connected with the formation of highly reactive cationic centers of type [L_2_M-H]^+^ [34,35,43].

Furthermore, quantum chemical modeling of the possible active sites governing the alkene polymerization also supports the proposed formation of the hydride intermediates and further participation in the reactions. For example, a DFT study of the initial stages of the methyl vinyl ether (MVE) polymerization which runs under the catalytic action of [Me_2_C(Cp)_2_Zr(Me)]^+^ and [Me_2_C(Cp)_2_Zr(H)]^+^ cations showed that it is the zirconium hydride which preferably initiates the polymer chain growth [44]. In Reference [43], devoted to the modeling of the propylene dimerization and oligomerization processes, the crucial role of the chlorine atom as a structural unit of the active Zr,Al- binuclear hydride intermediates was shown, and the efficiency of the molecular hydrogen as the activator that increases the rate and selectivity of the dimerization was revealed.

Our recent studies regarding the structure of the intermediates formed in the reaction of L_2_ZrCl_2_ with XAlBu^i^_2_ (X = H, Cl, Bu^i^) [32], as well as the complexes generated in zirconocene dihydride-ClAlR_2_ (R = Et, Bu^i^)-methylaluminoxane (MMAO-12) systems [45], showed that both systems L2ZrCl2-XAlBui2 and [Cp_2_ZrH_2_]_2_-ClAlR_2_ exhibit almost the same set of Zr,Al-hydride complexes. However, in the reaction of [Cp_2_ZrH_2_]_2_ with ClAlR_2_ (R = Et, Bu^i^), new bimetallic Zr,Zr-hydride complexes that can chemically bind to MAO were observed [45]. 

The goal of this research was to find the conditions for selective alkene dimerization in two catalytic systems: Cp_2_ZrCl_2_-(AlR_3_ or HAlBu^i^_2_ (R = Me, Et, Bu^i^))-MMAO-12, and [Cp_2_ZrH_2_]_2_-ClAlR_2_ (R = Me, Et, Bu^i^)-MMAO-12, and to reveal the structure of the zirconium hydride intermediates that initiate the alkene transformations.

## 2. Results

### 2.1. Study of 1-Alkene Transformations in Catalytic Systems Cp_2_ZrY_2_ (Y = Cl, H)-OAC-MMAO-12

#### 2.1.1. Activity and Chemoselectivity of System Cp_2_ZrCl_2_-(AlR_3_ or HAlBu^i^_2_)-MMAO-12 with Respect to Alkenes 

**Scheme 2:**
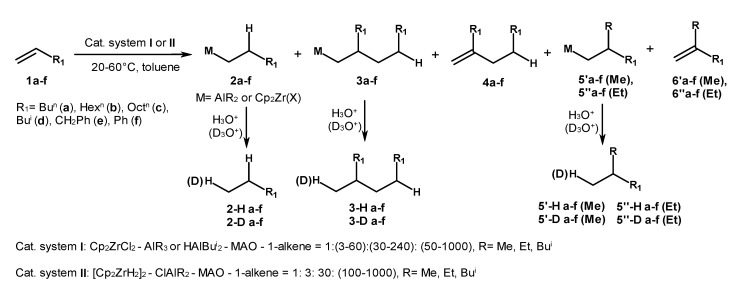
Alkene transformations in catalytic systems I or II.

On the first step of the catalytic experiments, we studied the effect of MMAO-12 on the activity and chemoselectivity of systems Cp_2_ZrCl_2_-XAlBu^i^_2_ (X = H, Bu^i^) that hydroaluminate the terminal alkenes in the absence of the activator [7]. As shown elsewhere, the catalytic system based on HAlBu^i^_2_ and zirconocene dichloride demonstrates low activity in the alkene hydroalumination (hydroalumination product (**2**)); this experiment was taken as a reference point (Scheme 2, Table 1 (entry 1)) [30,31,32]. Addition of 30–240 eq. of methylaluminoxane to the system does not affect notably the alkene conversion. For example, in the system L_2_ZrCl_2_-HAlBu^i^_2_-MMAO-12-1-octene at the ratio [Zr]:[Al]:[MAO]:[1-alkene] = 1:60:240:50, the conversion of 1-octene does not exceed 20% in 3 h (Table 1, (entry 4)). 

Unlike HAlBu^i^_2_, triisobutylaluminium is an effective reagent for 1-alkene hydrometalation catalyzed by Cp_2_ZrCl_2_ [30,32,46]. As another reference, the hydroalumination products after 3 h of the reaction were obtained at 83% yield (Table 1, entry 2) [32]. The addition of MMAO-12 increases the conversion of 1-alkene up to 98% due to the generation of both hydroalumination (**2**) and dimerization (**4**) products (Table 1, entry 3). Meanwhile, minor product **3**, the precursor of dimer **4**, was observed in this system due to the formation of the metal alkyl, which appeared after incorporation of the second alkene molecule into hydrozirconation product (Scheme 1). Rising the MMAO-12 content from 30 to 240 eq. increases the yield of 1-octene dimers (**4b**) from 5% to 40% (Table 1, entry 5). Similarly to the case with HAlBu^i^_2_, a decrease in the AlBu^i^_3_ concentration leads to the selective formation of the alkene dimers (97%) within 20 min of the reaction at 20 °C (Table 1, entry 6). A further increase in the temperature accelerates the reaction and helps to reduce the concentration of the catalytic system components without loss of both the chemoselectivity and the main product yield (entries 8,9). These results are consistent with the data obtained for catalytic system L_2_ZrCl_2_-AlBu^i^_3_-ClAlR_2_-MAO (1:20:(1–2):10, 0.05 mol% Zr, 60°C, 1–4 h), in which aluminum alkyl AlBu^i^_3_ and aluminum chloride ClAlR_2_ (R = Me, Et) activate the system that produces dimers with a high yield up to 94% [5,24,43]. 

Moreover, the conditions that we proved to be effective for the selective 1-octene dimerization were extended to 1-hexene treatment (entries 10,11). These conditions also provide high substrate conversion; however, the product distribution slightly changes due to the appearance of the hydrometalation (**2**) and alkylation (**6**) products, and the dimer yield becomes 89%–91%. 

Further, we studied the influence of different organoaluminum compounds (OAC) on the activity and chemoselectivity of the catalytic system; for this purpose, the isobutylalanes were replaced consequently by AlMe_3_ and AlEt_3_. The application of these OACs at 40 °C slightly reduces the alkene conversion to 91%–92% (entries 12,13). In the case of AlEt_3_, a decrease of the dimer yield to 68% was observed due to the increased fraction of the alkylated monomer **6** in the products (entry 13). The formation of **6** is possible through the alkene carbometalation product **5** which is generated by either methyl or ethyl zirconocenes formed during the stage of the alkyl-chloride exchange between Cp_2_ZrCl_2_ and either AlMe_3_ or AlEt_3_, respectively. It should be emphasized that for the systems like Cp_2_ZrCl_2_-AlMe_3_, these stages, finished by-product **6**, are very important since they provide zirconocene hydrides which are the source of dimers **4**. Rising of the temperature to 60 °C both increases the substrate conversion and narrows the selectivity towards the dimerization, regardless of the OAC nature (entries 14–21). Moreover, the amount of the substrate can be increased to 1000 eq. in the case of HAlBu^i^_2_ without loss of the dimer yield (98%, entry 22). 

As a result, we have established that the catalytic system based on HAlBu^i^_2_ provides the most selective dimerization (entries 14,18,22).

#### 2.1.2. Activity and Chemoselectivity of System [Cp_2_ZrH_2_]_2_-ClAlR_2_ (R = Me, Et, Bu^i^)-MMAO-12 with Respect to Alkenes

On the second step of the catalytic experiments, we studied the performance of system **II** [Cp_2_ZrH_2_]_2_-ClAlR_2_-MMAO-12 (R = Me, Et, Bu^i^) in the reaction with alkenes having various structures (**1a**–**f**). In all the experiments, the main product was dimer **4** (Scheme 2, Table 2).

Monitoring of the reaction for 3 h at the initial ratio of the reagents [Zr]:[Al]:[MAO]:[1-hexene] = 1:3:30:100 at 20 °C showed the presence of an induction period which duration significantly depends on the OAC structure. The longest induction period (60 min) was observed for ClAlMe_2_ (Figure S1a). For ClAlBu^i^_2_, the induction period decreases to 15 min (Figure S1c), and the yield of dimer **4** rises to 97% (Table 2, entry 6). A reaction in the presence of dimethyl- or diethylaluminum chlorides is accompanied by the formation of hydro- and carboalumination products with a yield of 22%–23% (Table 2, entries 3,4). As a result, systems based on [Cp_2_ZrH_2_]_2_, MMAO-12, and ClAlBu^i^_2_, compared to other ClAlR_2_, were found to be more active and selective. Diisobutylaluminum chloride also showed its effectiveness in the dimerization of other linear substrates: 1-octene and 1-decene. The yield of dimers **4b**,**c** obtained within 3 h at a temperature of 20 °C was 72%–98% (entries 7–18).

When the temperature is elevated to 40 °C, the reaction accelerates, and the induction period vanishes. The yield of 1-hexene and 1-octene dimers reaches 73%–91% in 15 min (entries 19–24). It should be noted that, in this case, the dependency of the system activity on the OAC nature disappears. Moreover, under these conditions, the conversion of 4-methyl-1-pentene (**1d**) is over 97%, and the yield of the dimerization products is 95% (entry 25). The temperature increase to 60 °C selectively provides 1-hexene dimers at 91%–94% yield within 5 min of the reaction (lines 26–28). Increasing 1-alkene initial concentration to 500 equivalents does not affect the degree of its transformation at 60 °C, and the yield of the dimerization products remains sufficiently high (85%–90%) (entry 32). A significant reduction in the substrate conversion and dimer yield (65% and 57%, correspondingly) was observed only at 1000 equivalents of the alkene (entry 33).

Moreover, the relative amount of MMAO-12 in the catalytic system also affects the activity only up to a certain level. The increase in the MMAO-12 content to 60 eq. (at 20 °C) accelerates the reaction and provides the dimers with a yield of 86% (entries 34–36). Further increase of MMAO-12 content up to 120 eq. does not change both the catalytic activity and the dimerization product yield (entry 37). 

It was established that aryl-substituted alkenes **1e** and **1f** can undergo the dimerization at an elevated temperature of 100 °C. Within 60 min, the dimers were obtained with yields of 61% and 58%, respectively. However, the proximity of the Ar group to the double bond leads to a loss of the reaction regioselectivity. The ratio of the regioisomers of the styrene dimer head-to-tail to tail-to-tail reaches 1.23:1 (entry 39).

Finally, the catalytic systems consisting of zirconocene dihydride, dialkylaluminum chlorides, and MMAO-12, work just as well as the systems based on zirconocene dichloride. Therefore, to identify the intermediates responsible for the alkene dimerization in both systems, we studied the structure and activity of the hydride complexes by the means of NMR spectroscopy.

### 2.2. NMR Study of Hydride Intermediate Structures in Systems Cp_2_ZrY_2_ (Y = Cl, H)-OAC-MMAO-12

**Scheme 3:**
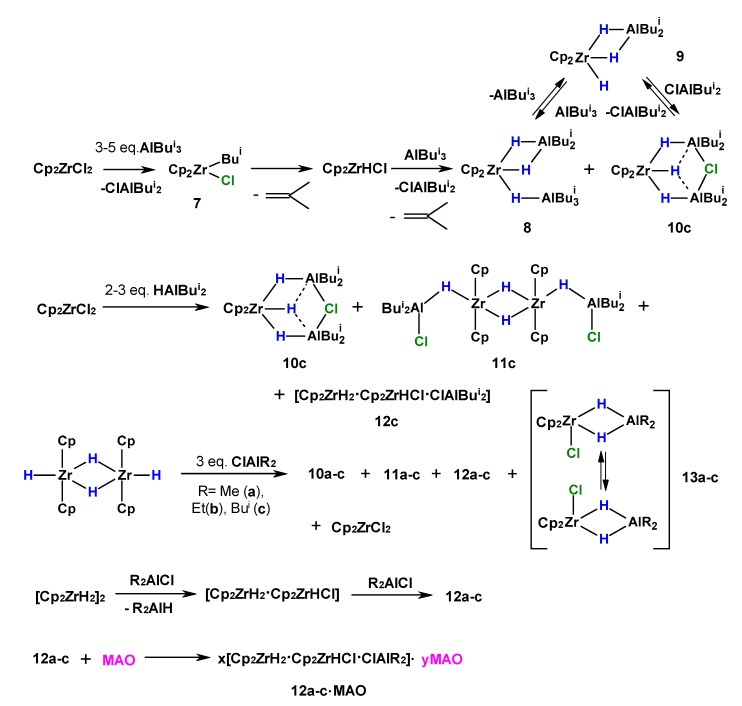
Reaction of Cp_2_ZrCl_2_ and [Cp_2_ZrH_2_]_2_ with OACs and MMAO-12.

**Figure 1:**
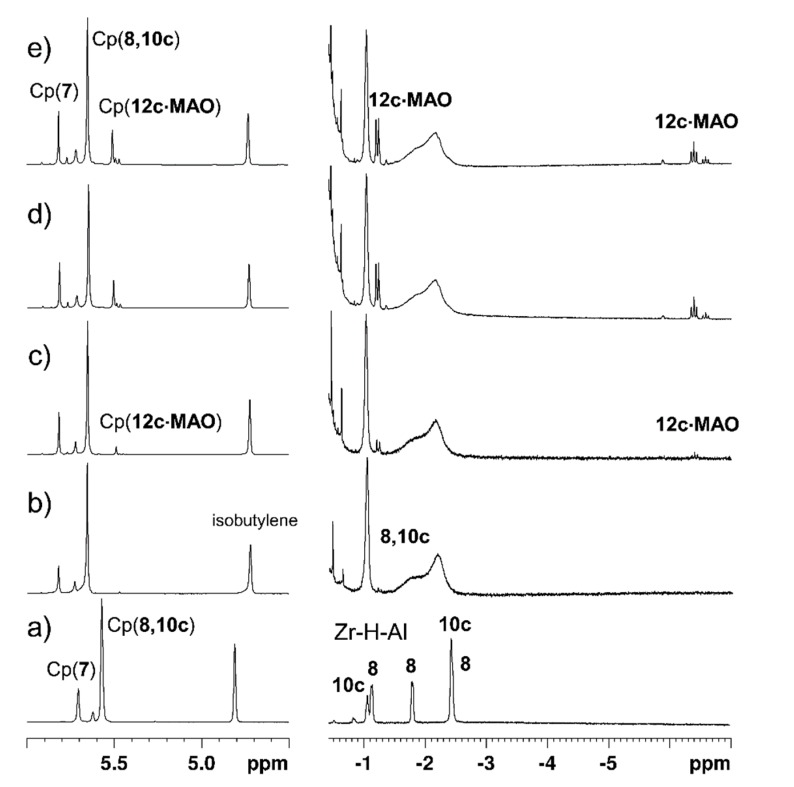
^1^H NMR of system Cp_2_ZrCl_2_-AlBu^i^_3_-MMAO-12 in C_7_D_8_ (T = 25 °C, intensity of upfield signals is increased): (**a**) [Zr]:[Al]:[MAO] = 1:5:0, T = −33 °C [32]; (**b**) [Zr]:[Al]:[MAO] = 1:5:0 [32]; (**c**) [Zr]:[Al]:[MAO] = 1:5:1.5; (**d**) [Zr]:[Al]:[MAO] = 1:5:5; (**e**) [Zr]:[Al]:[MAO] = 1:5:12.

Earlier, we showed that the system Cp_2_ZrCl_2_-XAlBu^i^_2_ can generate certain bimetallic hydride complexes; among them, the most active species in the hydroalumination reaction are the ones which have an open Zr-H bond [32]. The NMR study showed that in the Cp_2_ZrCl_2_ reaction with AlBu^i^_3_ (1:5), alkyl chloride complex **7** is produced and then transformed into complexes **8** and **10c**, both undergoing intermolecular exchange via intermediate **9** [30,32] (Scheme 3, Figure 1). In this work, the introduction of MMAO-12 into the reaction mixture, in the ^1^H NMR spectra, gives rise to both a doublet at −1.22 ppm (J = 17.6 Hz) and a triplet at −6.40 ppm (J = 17.6 Hz) which appear to be correlated in the COSY HH spectrum (Figure S5). Moreover, those signals are connected with a singlet of the Cp-rings at 5.50 ppm. The ratio of the signal intensities 2 (Zr-H):1 (Zr-H):20 (Cp) indicates the presence of two ZrCp_2_ fragments in the molecule. With an increase in the MMAO-12 concentration, additional doublet at −1.23 ppm and triplet at −6.59 ppm are observed; also, binary Cp-ring signals appear in the downfield part of the spectrum at 5.46 and 5.48 ppm. Taking into account these data, as well as the results obtained earlier [45], the structure of these complexes was assigned as **12** and is presented in Scheme 3. As we have previously shown [45], this type of complexes is formed in the reaction of [Cp_2_ZrH_2_]_2_ with ClAlR_2_ (R = Et, Bu^i^). In intermediates **12a–c**, there is no intermolecular exchange with hydride atoms; this indicates higher stability of these structures compared to complexes **8** and **10c**. 

The reaction of Cp_2_ZrCl_2_ with HAlBu^i^_2_ is accompanied by the formation of the known complexes **10c** [29,30,32] and **11c** [30,31]. With reduced HAlBu^i^_2_ content down to 2–3 equivalents (when the initial Cp_2_ZrCl_2_ remains unreacted), complex **12c** becomes observable; moreover, the complex is formed in this system even in the absence of MMAO-12 (Figures S7 and S8). Similar to the system with AlBu^i^_3_, addition of MMAO-12 increases the relative amount of complex **12c**, and a new set of signals similar to **12c** appear in the NMR spectrum. This phenomenon is possible due to the exchange reaction with AlMe_3_ contained in MMAO-12, as we described earlier [45].

**Figure 2:**
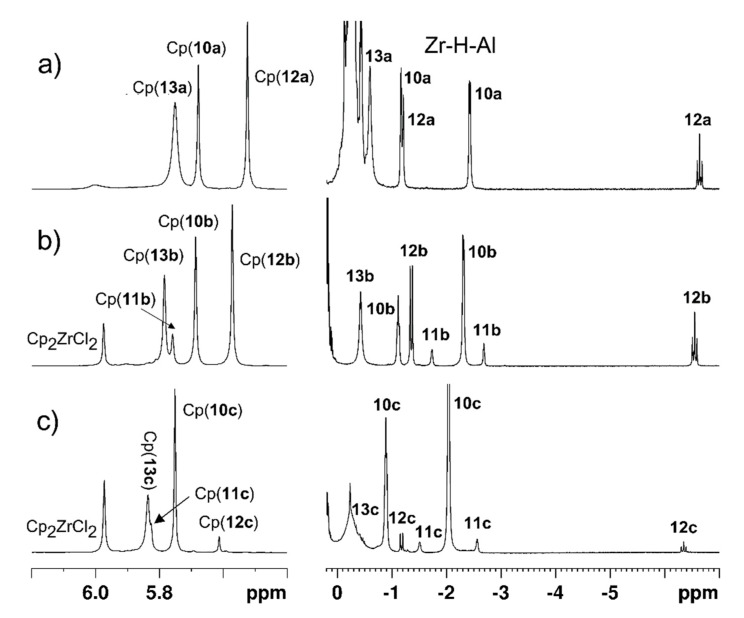
^1^H NMR of systems [Cp_2_ZrH_2_]_2_-ClAlR_2_ in C_7_D_8_ (T = 26 °C, intensity of upfield signals is increased): (**a**) ClAlMe_2_; (**b**) ClAlEt_2_ [45]; (**c**) ClAlBu^i^_2_ [45].

Zirconocene dihydride reacts with ClAlMe_2_ in a ratio of 1:3 providing a mixture of complexes: **10a**, **12a**, **13a**, and Cp_2_ZrCl_2_ (Scheme 3, Figure 2a, Table 3). The structure of complex **10a** was identified by analogy with **10b**,**c** [45]. A pair of signals at −0.60 ppm (Zr-H-Al) and 5.75 ppm (Cp) having an intensity ratio of 2:10 were assigned to complex **13a** [33]. The broadened nature of the signals belonging to the hydride atom and cyclopentadienyl rings indicates the participation of **13a** in the intermolecular exchange; a possible reaction pathway is shown in Scheme 3. Complex **12a** is characterized by both triplet and doublet signals at −6.64 ppm (J = 17.6 Hz) and −1.19 ppm (J = 17.6 Hz); these signals relate to that of Cp-rings at 5.52 ppm as 1:2:20. Probably, this complex is a result of the replacement of one hydride atom in the zirconocene dihydride dimer with chlorine. The dialkylaluminum hydride, produced as a result of the chloride-hydride exchange, reacts with Cp_2_ZrH_2_ and ClAlR_2_ and provides trihydride complexes **10a–c**.

**Figure 3:**
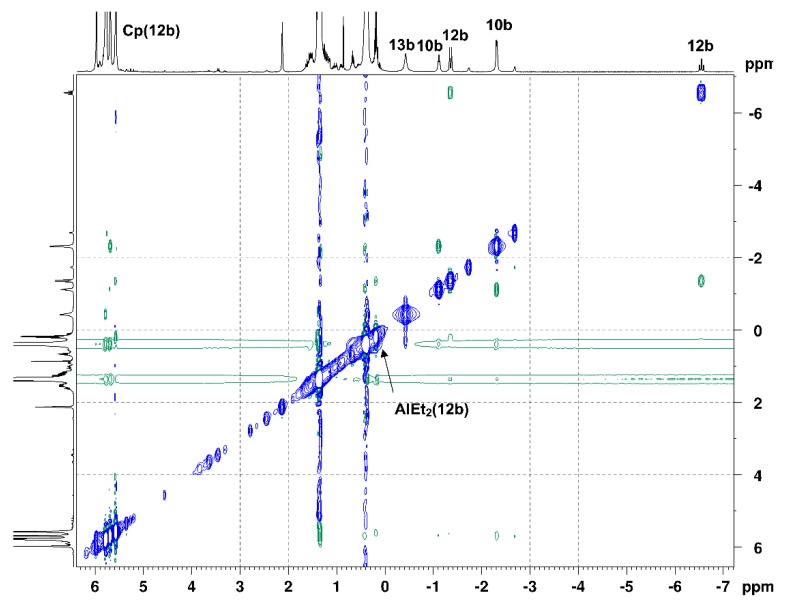
NOESY of [Cp_2_ZrH_2_]_2_-ClAlEt_2_ system (1:3) in C_7_D_8_.

Assessment of the NOESY spectrum for **12b** opened the possibility to clarify the structure of complexes **12a–c**. The spectrum exhibits cross-peaks between the signals of Cp- ring protons and hydride atoms with a quartet signal of ethyl group protons at 0.19 ppm (Figure 3); the relative intensity of the signals indicate the presence of no more than one ClAlEt_2_ molecule in the structure of **12b**.

**Figure 4:**
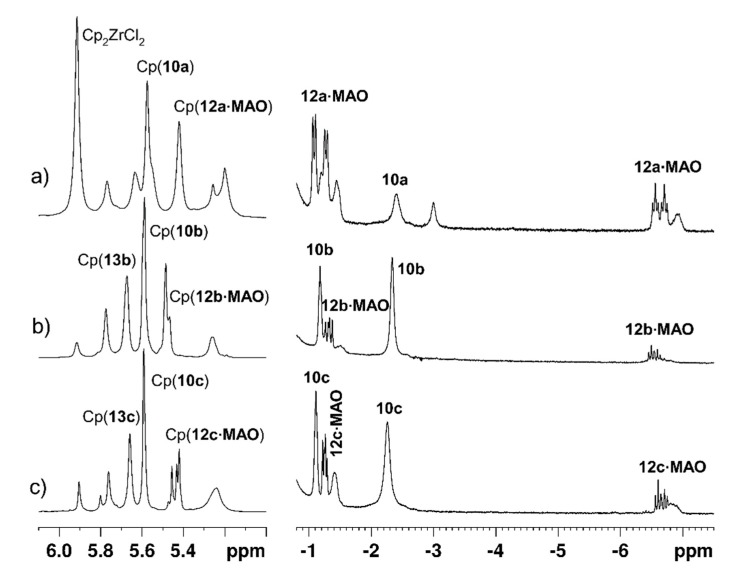
^1^H NMR of systems [Cp_2_ZrH_2_]_2_-ClAlR_2_-MMAO-12 in C_7_D_8_ (T = 26 °C, intensity of upfield signals is increased): (**a**) ClAlMe_2_, [Zr]:[Al]:[MAO] = 1:1.5:3; (**b**) ClAlEt_2_, [Zr]:[Al]:[MAO] = 1:3:6 [45]; (**c**) ClAlBu^i^_2_, [Zr]:[Al]:[MAO] = 1:3:8 [45].

After the addition of MMAO-12 to the Cp_2_ZrH_2_-ClAlMe_2_ system at a ratio [Zr]:[ClAlR_2_]:[MAO] = 1:3: (6–12), new upfield signals of the hydride atoms and protons of the cyclopentadienyl rings belonging to adducts of complexes **12a** with MMAO-12 (Figure 4a) were observed. In the case of complex **12a**, the ^1^H NMR spectrum exhibited both triplet and doublet signals at −6.56 and −1.08 ppm, respectively, as well as broadened signals at −6.94 and −1.43 ppm. These signals correspond to a broad peak of cyclopentadienyl rings at 5.11–5.33 ppm. Within a few minutes, the opacity of the homogeneous solution and separation of a heavy phase was detected. This corresponds to the previously observed effect for **12b**,**c** [45]. As follows from the NOESY experiment (Figure S17), adducts **12a∙MAO** show a negative NOE effect intrinsic to macromolecular compounds [47,48]. The signal broadening could be attributed to the high molecular weight of the particles. The substantial difference in diffusion coefficients between MMAO-12 derivatives (Figure S15) may indicate a large increase in the molecular weight and particle volume in the course of a heavy fraction formation. The latter is possible in the case of the intermolecular binding of MMAO-12 oligomers by the complexes. As a result, it was found that among a large set of bimetallic hydride complexes, only complexes **12a**–**c** were able to bind chemically to MMAO-12. 

Probably, the ability of the complexes to bind with the activator depends on the dynamic stability of the structures, and on the possibility to replace the organoaluminum fragment in the molecules. Moreover, the observation of adducts formed via covalent binding with methylaluminoxane suggests the presence of a considerable number of accessible three-coordinated Lewis-acidic aluminum centers in the activator. Since MMAO-12 acts similarly to trimethylaluminum or ClAlR_2_, which can substitute AOC in the complexes **12a**–**c**, it can be assumed that there is a sufficient number of OAlMe_2_ groups in the methylaluminoxane structure [49,50,51,52].

**Scheme 4:**
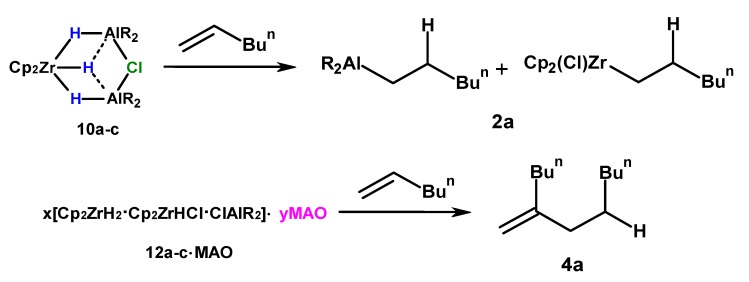
Reaction of complexes **10a**–**c** and **12a**–**c∙MAO** with 1-hexene.

**Figure 5:**
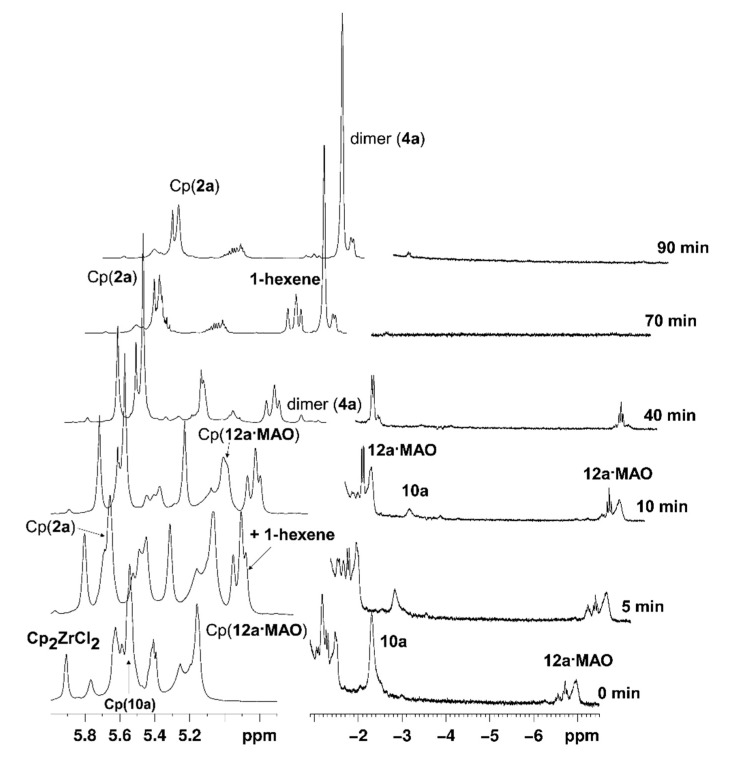
^1^H NMR monitoring of 1-hexene transformation in the system [Cp_2_ZrH_2_]_2_-ClAlMe_2_-MMAO-12 in C_7_D_8_ (T = 26 °C, intensity of upfield signals is increased).

Using the complexes obtained in the [Cp_2_ZrH_2_]_2_-ClAlBu^i^_2_ system as an example, we showed that the addition of 1-hexene to a solution containing a mixture of complexes **10c**–**13c** in the absence of MMAO-12 is accompanied with the appearance of hydrometalation products **2a**: zirconocene alkyl chloride and aluminum alkyl (Scheme 4); their identification was carried out on the base of NMR data [30] and mass spectrometry of the deuterolysis products. For this reaction, complexes **10c** and **11c** interacted first, whereas **12c** was no longer observed in the spectra only after 4 h of the process (Figure S19). As follows from Figure 5, in the presence of MMAO-12, when the system contains MAO-adducts along with the complexes **10c**, **12c**, **13c**, complex **10** is consumed quickly, providing hydrometalation products **2a**. The formation of the dimers is accompanied by the vanishing of the MAO- adducts of complex **12**; especially their heavy fraction is consumed first.

## 3. Discussion

Our study of the catalytic system Cp_2_ZrCl_2_-OAC-MMAO-12 proved the ability of this type of system to dimerize 1-alkenes with high chemoselectivity to give head-to-tail products with a vinylidene moiety > C=CH_2_ [5,9,10,11,12,24,53]. The other Ti subgroup metal compounds, for example, postmetallocene Zr and Hf complexes with [ONNO]-type amine bis(phenolate) ligands, activated by B(C_6_F_5_)_3_, catalyze the 1-hexene oligomerization with the same regioselectivity [54]. Among these complexes, hafnium catalysts showed the best selectivity towards the dimers (up to 97%); moreover, the molecular weight distribution of the oligomers, obtained in Hf-catalyzed reactions, do not correspond to a typical Schulz−Flory regularity. Similar zirconium complexes with aryl-substituted [OSSO]-type bis(phenolate) ligands on the trans-cyclooctanediyl platform, activated by dried modified methylaluminoxane (dMMAO), catalyze 1,2-regioselective oligomerization of 1-hexene at relatively low catalyst loadings and produce preferably head-to-tail dimer. The use of other transition metal complexes leads, first of all, to changes in the reaction regioselectivity. For example, the systems based on Fe [55] and Co [56,57] complexes and Al- coactivators provide linear head-to-head dimers, whereas W catalysts [58] give predominantly methyl- and dimethyl-branched tail-to-tail products. In these studies, the participation of the transition metal hydride complexes as active centers is assumed as given. 

However, in the literature, information regarding the direct evidence of the alkene dimerization under the action of metal hydrides is very limited [59,60]. For example, it was shown that the binuclear hydride complex [(Ind’)_2_Y(*μ*-H)]_2_ catalyzes regio- and stereoselective homodimerization of various α-olefins at 80 °C, as well as head-to-head codimerization of styrene with other α-olefins [59]. In this work, we show that the activity and chemoselectivity of the systems based on zirconocene dichloride and dihydride are comparable to each other; this outlines the zirconium hydrides as the probable precursors of active intermediates of the dimerization reaction. 

As follows from the NMR study, Zr,Al-hydride clusters **10a**–**c**, even in the presence of MMAO-12 activator, give only hydrometalation products, while the dimerization pathway is determined by biszirconium hydride complexes **12a**–**c**. The comparison of the action of complexes **10** and **12** shows that the first stage of the reaction, as presented in Scheme 1, is the hydrometalation stage. Further, the process flow is possible in two directions: either transmetalation with the transfer of the alkyl fragment to aluminum or the introduction of a second alkene molecule into the hydrozirconation product with the formation of the dimer. Therefore, for the bimetallic Zr,Al- hydride complexes **10a**–**c**, the processes of the alkene hydrometalation and subsequent transmetalation proceed in the most rapid manner. Indeed, as shown by quantum chemical modeling [61], the limiting stages of the reaction are either the stage of the alkene incorporation into zirconocene hydrochloride (ΔG^≠^ = 10.4 kcal/mol), or the stages of Zr−Cl and Zr−H bridge bond breaking in Zr,Al-bimetallic complexes (17.8 kcal/mol and 19.8 kcal/mol) before the alkene coordination. The transmetalation stages have insignificant or even no activation barriers. Complex **12** is also, apparently, like **10**, capable for the alkene hydrometalation; however, its activation with methylaluminoxane gives active species which selectively provide dimers. Specifically, the stages of the consecutive incorporation of the two alkene molecules and the chain termination through *β*-H elimination proceed fast, and without the direct involvement of the organoaluminum fragment which, when located close to hydride atoms, would facilitate chain transfer to aluminum. 

In broad understanding, this case can be considered as an example of biszirconium catalysis similar to the alkene polymerization in the presence of Group 4 bimetallic complexes [62,63,64]. Further development of this work into establishing the activation mechanism of the biszirconium hydride complexes can be fruitful in uncovering the reasons for the selective formation of alkene di- and oligomers.

## 4. Materials and Methods 

^1^Н and ^13^C NMR spectra were recorded on a Bruker AVANCE-400 spectrometer (400.13 MHz (^1^H), 100.62 MHz (^13^C)) (Bruker, Rheinstetten, Germany). As the solvents and the internal standards, C_7_D_8_ (toluene-d_8_) and CDCl_3_ were employed. 1D and 2D NMR spectra (COSY HH, HSQC, HMBC, NOESY) were recorded using standard Bruker pulse sequences. 1D and 2D DOSY spectra were obtained using ledbpgp2s pulse program (LED with bipolar gradient pulse pair, 2 spoil gradients). The experiments were carried out at 23–25 °C and temperature stabilization accuracy within 0.1 °C. The acquisition parameters for the diffusion experiments were δ = 1 ms, Δ = 0.1–0.2 s. 

The yields of compounds **2**–**5** were determined from the yields of hydrolysis and deuterolysis products, which were calculated relative to the amount of the initial olefin. The products were analyzed using a gas chromatograph mass spectrometer GCMS-QP2010 Ultra (Shimadzu, Tokyo, Japan) equipped with the GC-2010 Plus chromatograph (Shimadzu, Tokyo, Japan), TD-20 thermal desorber (Shimadzu, Tokyo, Japan), and an ultrafast quadrupole mass-selective detector (Shimadzu, Tokyo, Japan).

### 4.1. General Procedures

All operations for organometallic compounds were performed under argon according to the Schlenk technique. The zirconocene dichloride was prepared using the standard procedure from ZrCl_4_ (99.5%, Merck, Darmstadt, Germany) [65]. The synthesis of [Cp_2_ZrH_2_]_2_ from Cp_2_ZrCl_2_ was performed as described previously [29,30,31]. The solvents (benzene, toluene) were distilled from AlBu^i^_3_ immediately before use. Commercially available HAlBu^i^_2_ (99%, Merck, Darmstadt, Germany), ClAlEt_2_ (97%, Strem, Kehl, Germany), AlBu^i^_3_ (95%, Strem, Kehl, Germany), AlEt_3_ (98%, Merck, Darmstadt, Germany), AlMe_3_ (97%, Merck, Darmstadt, Germany) MMAO-12 (7% Al in toluene, Merck, Darmstadt, Germany) were involved into the reactions. CAUTION: the pyrophoric nature of aluminum alkyl and hydride compounds require special safety precautions in their handling. Terminal alkenes 1-hexene (97%, Acros, Geel, Belgium), 1-octene (99%, Acros, Geel, Belgium), 1-decene (95%, Acros, Geel, Belgium), 4-methyl-1-pentene (97%, Acros, Geel, Belgium), allylbenzene (98%, Acros, Geel, Belgium), styrene (99%, Fisher, Hampton, NH, USA) were used as received. NMR data, mass spectra of the obtained dimers **4a**–**f** correspond to the data, presented in Refs. [5,10,11,66].

### 4.2. Reaction of Cp_2_ZrCl_2_ with OAC, MMAO-12 and 1-Alkene

A flask with a magnetic stirrer was filled under argon with 0.0342 mmol (10 mg) of Cp_2_ZrCl_2_, 0.103–2.05 mmol of OAC (AlMe_3_, AlEt_3_, HAlBu^i^_2_, AlBu^i^_3_), 0.925–8.2 mmol of MMAO-12 and 1.71–34.3 mmol of 1-alkene. The reaction was carried out with stirring at different temperatures 20, 40 and 60 °C. After 5, 10, 15, 30, 45, 60 min, samples (0.1 mL) were syringed into tubes filled with argon, and the samples were decomposed with 10% HCl or DCl at 0 °C. Products were extracted with CH_2_Cl_2_, and the organic layer was dried over Na_2_SO_4_. The yield of products was determined by GC-MS. 

### 4.3. Reaction of [Cp_2_ZrH_2_]_2_ with ClAlR_2_, MMAO-12 and 1-Alkene

A flask with a magnetic stirrer was filled under argon with 0.022 mmol (10 mg) of [Cp_2_ZrH_2_]_2_, 0.135 mmol of ClAlR_2_, 0.135–5.4 mmol of MMAO-12 and 4.5–45 mmol of 1-alkene. The reaction was carried out with stirring at different temperatures 20, 40 and 60 °C. After 5, 10, 15, 30, 60, 90, 120, 150, 180, 240 and 360 min, samples (0.1 mL) were syringed into tubes filled with argon, and the samples were decomposed with 10% HCl or DCl at 0 °C. Products were extracted with CH_2_Cl_2_, and the organic layer was dried over Na_2_SO_4_. The yield of products was determined by GC-MS.

### 4.4. NMR Study of the Reaction of Cp_2_ZrCl_2_ with XAlBu^i^_2_ (X = H, Bu^i^) and MMAO-12

**Method A.** An NMR tube was charged with 0.034 mmol (10 mg) of Cp_2_ZrCl_2_ and 0.5 mL of C_7_D_8_ in an argon-filled glovebox. The tube was cooled to 10 °C and 0.051–0.185 mmol of XAlBu^i^_2_ (X = H, Bu^i^) was added dropwise. The mixture was stirred and the formation of complexes **10**–**13** was monitored by NMR at room temperature. Further addition of 0.051–0.41 mmol of MMAO-12 provided a separation of the reaction media into two fractions. **Method B.** An NMR tube was charged with 0.051–0.41 mmol of MMAO-12, 0.5 mL of C_7_D_8_ and 0.051–0.185 mmol of XAlBu^i^_2_ (X = H, Bu^i^) in an argon-filled glovebox. The tube was cooled to 10 °C and 0.034 mmol of Cp_2_ZrCl_2_ was added. The mixture was stirred and the formation of complexes was monitored by NMR at room temperature.

### 4.5. NMR Study of the Reaction of [Cp_2_ZrH_2_]_2_ with ClAlR_2_ (R=Me, Et, Bu^i^) and MMAO-12

**Method A.** An NMR tube was charged with 0.022 mmol (10 mg) of [Cp_2_ZrH_2_]_2_ and 0.5 mL of C_7_D_8_ in an argon-filled glovebox. The tube was cooled to 10 °C and 0.044–0.135 mmol of ClAlR_2_ was added dropwise. The mixture was stirred and the formation of complexes **10**–**13** was monitored by NMR at room temperature. Further addition of 0.067–0.54 mmol MMAO-12 provided a separation of the reaction media into two fractions. **Method B.** An NMR tube was charged with 0.067–0.54 mmol of MMAO-12, 0.044–0.135 mmol of ClAlEt_2_ and 0.5 mL of C_7_D_8_ in an argon-filled glovebox. The tube was cooled to 10 °C and 0.022 mmol (10 mg) of [Cp_2_ZrH_2_]_2_ was added. The mixture was stirred and the formation of complexes was monitored by NMR at room temperature.

## 5. Conclusions

In summary, our studies on the alkene transformations under the action of MAO-activated systems Cp_2_ZrCl_2_-(AlR_3_ or HAlBu^i^_2_) and [Cp_2_ZrH_2_]_2_-ClAlR_2_ (R = Me, Et, Bu^i^) show their capability to provide dimeric products with high yield and selectivity at the low content of the OAC and methylaluminoxane. Parallel studies on the systems have shown a deep similarity both in the catalytic performance and intermediate composition. As a result of the NMR studies, among all the intermediates considered, we proved for the first time that new Zr,Zr- hydride complexes having type x[Cp_2_ZrH_2_∙Cp_2_ZrHCl∙ClAlR_2_]∙yMAO appear to be specifically responsible for the alkene dimerization. Further study of the reaction mechanism will uncover the process of activation of biszirconium complexes by methylaluminoxane, and this will explain the unique selectivity of these intermediates in the dimerization pathway. 

## Figures and Tables

**Table 1:** Catalytic activity and chemoselectivity of system **I** Cp_2_ZrCl_2_-OAC-MMAO-12 in the reaction with 1-alkene.

Entry	1-Alkene	OAC	[Zr]:[Al]:[MAO]:[1-alkene]	T, °C	Time, min	Alkene Conversion, %	Product yield, ^1^ %				
							2-D	3-D	4	5-D	6
1	1-octene (**1b**)	HAlBu^i^_2_ [32]	1:60:0:50	20	180	11	11	-	-	-	-
2		AlBu^i^_3_ [32]				83	83	-	-	-	-
3		AlBu^i^_3_	1:60:30:50	20	180	98	91	2	5	-	-
4		HAlBu^i^_2_	1:60:240:50	20	180	20	20	-	-	-	-
5		AlBu^i^_3_				94	51	3	40	-	-
6		AlBu^i^_3_	1:3:30:50	20	20	99	1	1	97	-	-
7			1:3:30:100	20	30	99	-	<1	98	-	<1 (**6′**) ^8^
8		HAlBu^i^_2_	1:3:30:100	40	10	99	-	3	96	-	-
9		AlBu^i^_3_				99	-	1	98	-	-
10	1-hexene (**1a**)	HAlBu^i^_2_	1:3:30:100	40	15	99	5	1	91	-	2 (**6′**) ^8^
11		AlBu^i^_3_				99	4	1	89	-	3 (**6′**) ^8^
12		AlMe_3_				91	-	-	87	2 (**5′**)	2 (**6′**)
13		AlEt_3_				92	2	3	68	-	6 (**6′**) ^8^ 12 (**6″**)
14		HAlBu^i^_2_	1:3:30:250	60	5	98	1	1	94	-	2 (**6′**) ^8^
15		AlBu^i^_3_				99 ^2^	1	1	88	-	4 (**6′**) ^8^
16		AlMe_3_				99 ^3^	1	1	85	-	3 (**6′**)
17		AlEt_3_				96 ^3^	1	1	85	-	1 (**6′**) ^8^
18		HAlBu^i^_2_	1:3:30:500	60	5	98	-	-	97	-	-
19		AlBu^i^_3_				98	1	1	93	-	1 (**6′**) ^8^
20		AlMe_3_				99 ^4^	1	1	87	-	5 (**6′**)
21		AlEt_3_				98	1	1	96	-	<1 (**6′**) ^8^
22		HAlBu^i^_2_	1:3:30:1000	60	15	99	-	-	98	-	-
23					30	83 ^5^	-	-	78	-	-
24					60	86 ^5,6^	<1	-	83	-	<1 (**6′**) ^8^
25		AlBu^i^_3_			15	94 ^6^	-	-	92	-	1 (**6′**) ^8^
26		AlMe_3_			10	92	2	-	84	-	6 (**6′**)
27		AlEt_3_			60	65 ^7^	-	-	64	-	<1 (**6″**)

^1^ Determined by GC-MS of deuterolysis products. ^2^ Formation of trimers up to 5% is observed. ^3^ Trimers—8%. ^4^ Trimers—5%. ^5^ 2000 eq. of 1-hexene were taken; trimers up to 5% are observed. ^6^ Trimers—1%. ^7^ 10 eq. of AlEt_3_ were taken. ^8^ Product 6′ was formed due to the presence of residual AlMe_3_ in MMAO-12.

**Table 2:** Catalytic activity and chemoselectivity of system **II** [Cp_2_ZrH_2_]_2_-ClAlR_2_-MMAO-12 in the reaction with terminal alkenes (all ratios are given with respect to monomeric Cp_2_ZrH_2_).

Entry	1-Alkene	ClAlR_2_	[Cp_2_ZrH_2_]: [Al]: [MAO]:[1-alkene]	T, °C	Time, min	Alkene Conversion, %	Product Yield, ^1^ %				
							2-D	3-D	4	5-D	6
1	1-hexene **(1a)**	ClAlMe_2_	1:3:30:100	20	60	32	2	7	15	1 (**5′**)	7 (**6′**)
2					180	99	2	6	80	2 (**5′**)	9 (**6′**)
3		ClAlEt_2_			60	75	2	4	47	-	22 (**6″**)
4					180	99	2	5	69	-	23 (**6″**)
5		ClAlBu^i^_2_			60	79	1	5	73	-	-
6					180	99	1	2	97	-	-
7	1-octene (**1b**)	ClAlMe_2_			60	25 ^2^	1	4	15	-	4 (**6′**)
8					180	>99 ^2^	4	10	72	3	9 (**6′**)
9		ClAlEt_2_			60	73 ^2^	2	13	46	-	11 (**6′**) ^4^
10					180	>99 ^2^	3	9	76	2 (**5′**) ^4^	9 (**6′**) ^4^
11		ClAlBu^i^_2_			60	85	2	12	60	-	10 (**6′**) ^4^
12					180	>99 ^2^	3	9	77	2 (**5′**) ^4^	8 (**6′**) ^4^
13	1-decene (**1c**)	ClAlMe_2_			60	<5	-	2	3	-	-
14					180	>99	3	6	81	1 (**5′**)	8 (**6′**)
15		ClAlEt_2_			60	68	4	10	28	5 (**5″**)	21 (**6″**)
16					180	>99	6	4	76	2 (**5′**) ^4^	12 (**6′**) ^4^
17		ClAlBu^i^_2_			60	73	4	7	59	-	2 (**6′**) ^4^
18					180	>99	4	7	87	-	2 (**6′**) ^4^
19	1-hexene (**1a**)	ClAlMe_2_		40	15	96	4	3	82	-	10 (**6′**)
20		ClAlEt_2_			15	98	2	3	83	-	11 (**6′**) ^4^
21		ClAlBu^i^_2_			15	>99	4	2	86	-	9 (**6′**) ^4^
22	1-octene (**1b**)	ClAlMe_2_			15	83	4	3	73	-	4 (**6′**)
23		ClAlEt_2_			15	89	4	3	79	-	1 (**6′**) ^4^ 2 (**6″**)
24		ClAlBu^i^_2_			15	98	5	-	91	-	2 (**6′**) ^4^
25	4-methyl-1-pente-ne (**1d**)	ClAlBu^i^_2_			30	>99	1	1	95	-	2 (**6′**) ^4^
26	1-hexene (**1a**)	ClAlMe_2_		60	5	99	1	1	91	-	6 (**6′**)
27		ClAlEt_2_			5	99	1	2	92	-	5 (**6′**) ^4^
28		ClAlBu^i^_2_			5	99	-	2	94	-	3 (**6′**) ^4^
29		ClAlMe_2_	1:3:30:250	60	5	94	1	1	85	-	6 (**6′**)
30		ClAlEt_2_			5	98 ^3^	1	2	90	-	5 (**6″**)
31		ClAlBu^i^_2_			5	98	1	2	87	-	3 (**6′**) ^4^
32		ClAlBu^i^_2_	1:3:30:500	60	30	95	1	2	87	-	5 (**6′**) ^4^
33		ClAlBu^i^_2_	1:3:30:1000	60	360	65 ^2^	1	2	57	-	3 (**6′**) ^4^
34		ClAlBu^i^_2_	1:3:3:100	20	240	41	2	3	35	-	-
35		ClAlBu^i^_2_	1:3:12:100	20	240	99	-	13	86	-	-
36		ClAlBu^i^_2_	1:3:60:100	20	15	>99	2	<1	81	2 (**5′**) ^4^	14 (**6′**) ^4^
37		ClAlBu^i^_2_	1:3:120:100	20	15	>99	1	2	80	1 (**5′**) ^4^	15 (**6′**) ^4^
38	Allylbenzene (**1e**)	ClAlBu^i^_2_	1:3:30:100	100	60	94	6	2	52 + 9 ^5^	-	25 (**6′**) ^4^
39	Styrene (**1f**)	ClAlBu^i^_2_			60	79 ^3^	8	-	32 + 26 ^5^	-	6 (**6′**) ^4^

^1^ Determined by GC-MS of deuterolysis products. ^2^ Formation of trimers up to 1%–2% is observed. ^3^ Trimers—8%. ^4^ Products **5′** or **6′** were formed due to the presence of residual AlMe_3_ in MMAO-12. ^5^ The formation of two regioisomers head-to-tail and tail-to-tail occurs.

**Table 3:** ^1^H and ^13^C NMR of complexes **10a**, **12a**, **13a**, **12a∙MAO** (δ, ppm, 400.13 MHz, C_7_D_8_, T = 27 °C).

Complex	δ_H_ Cp	δ_C_ Cp	δ_H_ Zr-H-Zr	δ_H_ Zr-H-Al	δ_H_ MAO
**10a**	5.68 (s, 10H)	104.74		−2.43 (d, 8.6 Hz, 2H) −1.19 (t, 8.6 Hz, 1H)	
**11a**	5.72 (s, 10H)	107.90		−2.55 (br.s, 1H) −1.60 (br.s, 1H)	
**12а**	5.52 (s, 20H)	108.00	−6.64 (t, 17.6 Hz, 1H) −1.19 (d, 17.6 Hz, 2H)		
**13a**	5.75 (br.s, 10H)	108.29 (br.)		−0.60 (br.s, 2H)	
**12a∙MAO**	5.42	107.88	−6.56 (t, 17.2 Hz, 1Н) −1.08 (d, 17.2 Hz, 2Н) −6.71 (t, 17.6 Hz, 1Н) −1.27 (d, 17.6 Hz, 2Н)		
**12a∙MAO (heavy phase)**	5.11–5.33	107.59 (br.)	−6.92 (br.t, 2H) −1.44 (br.d, 2H)		−0.63 ÷ −0.08

## Supplementary Materials

Supplementary Materials are available online.
