# Supplementary material for: Bimetallic Zr,Zr-Hydride Complexes in Zirconocene Catalyzed Alkene Dimerization
Source: Molecules. 2020 May 8;25(9):2216. doi: 10.3390/molecules25092216 (PMC7248859; doi:10.3390/molecules25092216)

Bimetallic Zr,Zr- Hydride Complexes in Zirconocene Catalysed Alkene Dimerization

Lyudmila V. Parfenova[1,*], Pavel V. Kovyazin[1], Almira Kh. Bikmeeva[1]

[1]*Institute of Petrochemistry and Catalysis of Russian Academy of Sciences, 141, Prospekt Oktyabrya, 450075 Ufa, Russia; kpv38@mail.ru (P.V.K.); almira.bikmeeva@gmail.com (A.K.B.)*
***Correspondence:** *luda_parfenova@ipc-ras.ru (L.V.P.)*

Supporting Information

Figure S1. Effect of OAC structure on product yield in the system $[Cp_2ZrH_2]_2$−$ClAlR_2$−MMAO-12− 1-hexene (1:3:30:100, 20°C)........ 3
Figure S2. ^{1}H NMR of system Cp_2ZrCl_2 − $AlBu^i_3$ (1:5) in C_7D_8 (240 K)........ 4
Figure S3. ^{1}H NMR of system Cp_2ZrCl_2 − $AlBu^i_3$ (1:5) in C_7D_8 (298 K). 4
Figure S4. ^{1}H NMR of system Cp_2ZrCl_2 − $AlBu^i_3$ – MMAO-12 (1:5:12) in C_7D_8 (298 K)........ 5
Figure S5. COSY HH of system Cp_2ZrCl_2 − $AlBu^i_3$ – MMAO-12 (1:5:12) in C_7D_8 (298 K)........ 5
Figure S6. NOESY of system Cp_2ZrCl_2 − $AlBu^i_3$ – MMAO-12 (1:5:12) in C_7D_8 (298 K)... 6
Figure S7. ^{1}H NMR of system Cp_2ZrCl_2–$HAlBu^i_2$–MMAO-12 in C_7D_8 (T= 298 K): a) [Zr]:[Al]:$[Al_{MAO}]$= 1:1.5:0; b) [Zr]:[Al]:$[Al_{MAO}]$= 1:1.5:1.5; c) [Zr]:[Al]:$[Al_{MAO}]$= 1:1.5:3........ 6
Figure S8. ^{1}H NMR of system Cp_2ZrCl_2 − $HAlBu^i_2$ (1:2) in C_7D_8........ 7
Figure S9. ^{1}H NMR of system Cp_2ZrCl_2 − $HAlBu^i_2$ – MMAO-12 (1:1.5:3) in C_7D_8........ 7
Figure S10. ^{1}H NMR of system $[Cp_2ZrH_2]_2$ − $ClAlMe_2$ (1:3) in C_7D_8........ 8
Figure S11. COSY HH of system $[Cp_2ZrH_2]_2$ − $ClAlMe_2$ (1:3) in C_7D_8........ 8
Figure S12. ^{13}C NMR of system $[Cp_2ZrH_2]_2$ − $ClAlMe_2$ (1:3) in C_7D_8........ 9
Figure S13. HSQC of system $[Cp_2ZrH_2]_2$ − $ClAlMe_2$ (1:3) in C_7D_8........ 9
Figure S14. ^{1}H NMR of system $[Cp_2ZrH_2]_2$ − $ClAlMe_2$ – MMAO-12 (1:3:6) in C_7D_8........ 10
Figure S15. DOSY of system $[Cp_2ZrH_2]_2$−$ClAlMe_2$–MMAO-12 (1:3:6) in C_7D_8 (T=299.3 K)........ 10
Figure S16. COSY of system $[Cp_2ZrH_2]_2$−$ClAlMe_2$–MMAO-12 (1:3:6) in C_7D_8........ 11
Figure S17. NOESY of system $[Cp_2ZrH_2]_2$−$ClAlMe_2$–MMAO-12 (1:3:6) in C_7D_8........ 11
Figure S18. ^{1}H NMR of system $[Cp_2ZrH_2]_2$ − $ClAlEt_2$ (1:3) in C_7D_8........ 12
Figure S19. NMR monitoring of system $[Cp_2ZrH_2]_2$− $ClAlBu^i_2$ – 1-hexene (1:2.6:(0.7-2.4)) in C_7D_8, intensity of upfield signals is increased........ 12

Figure S20. ^{13}C NMR of system $[Cp_2ZrH_2]_2$ − $ClAlBu^i_2$ – 1-hexene (1:2.6:(0.7-2.4)) in C_7D_8 (end of reaction).. 13

Figure S21. ^{13}C NMR of system $[Cp_2ZrH_2]_2$ − $ClAlMe_2$ – MMAO-12 – 1-hexene in C_7D_8 (end of reaction).. 13

GC-MS analysis of products... 14

Figure S22. Example of GC-MS of products obtained in the system Cp_2ZrCl_2 – $AlMe_3$ – MMAO-12– 1-hexene 14

Figure S1. Effect of OAC structure on product yield in the system $[Cp_2ZrH_2]_2$−$ClAlR_2$−MMAO-12− 1-hexene (1:3:30:100, 20°C):
(a) - $ClAlMe_2$; (b) - $ClAlEt_2$; (c) $ClAlBu^i_2$.

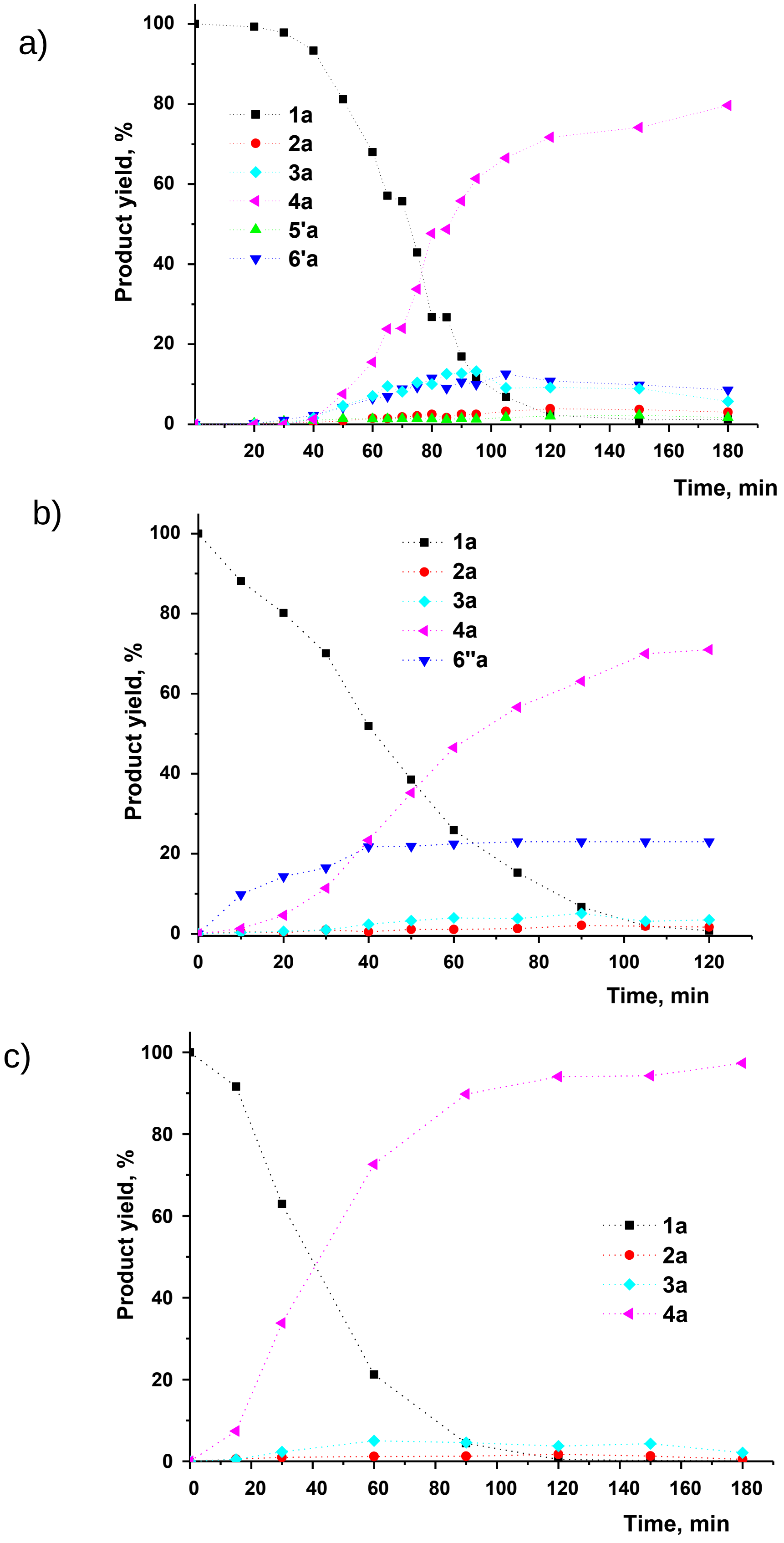

Figure S2. ^{1}H NMR of system Cp_2ZrCl_2 − $AlBu^i_3$ (1:5) in C_7D_8 (240 K).

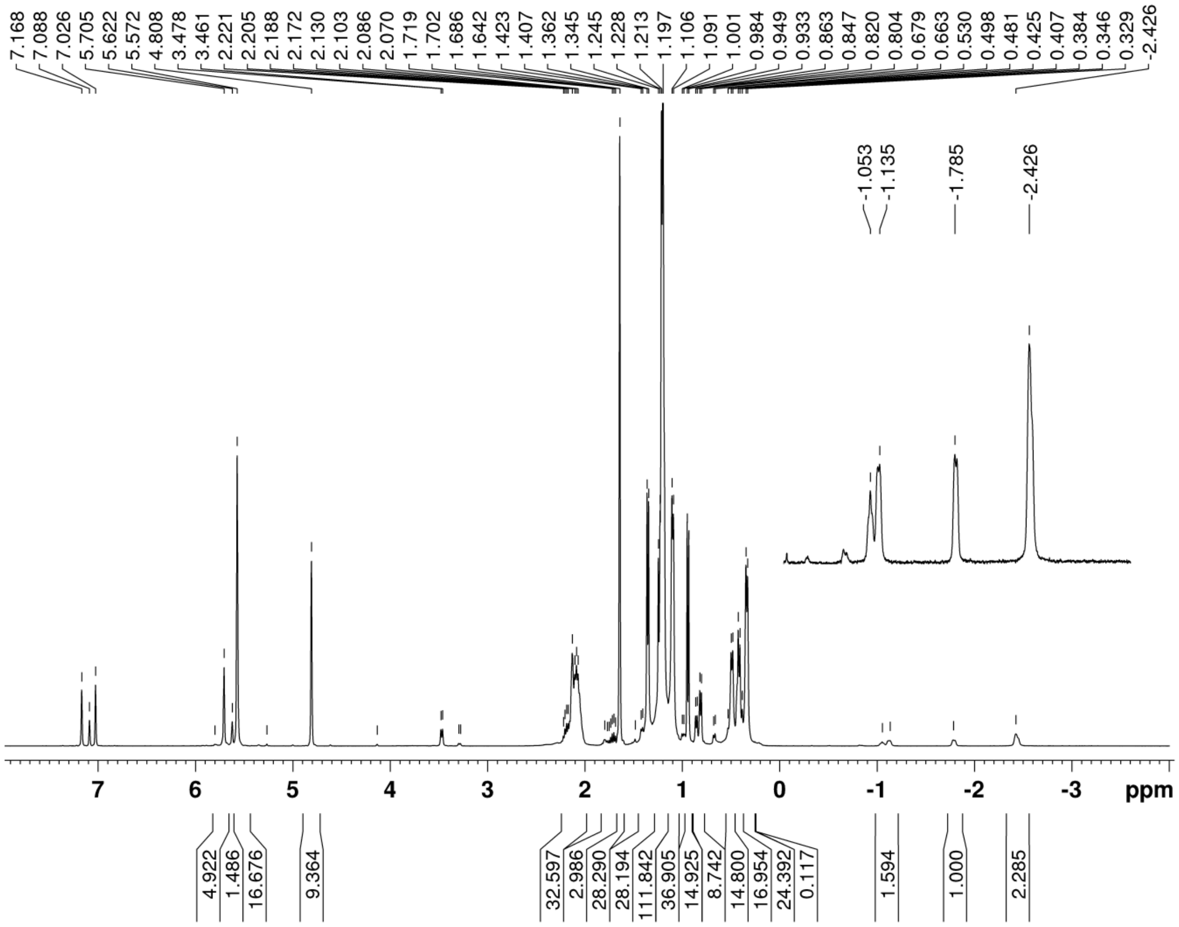

Figure S3. ^{1}H NMR of system Cp_2ZrCl_2 − $AlBu^i_3$ (1:5) in C_7D_8 (298 K).

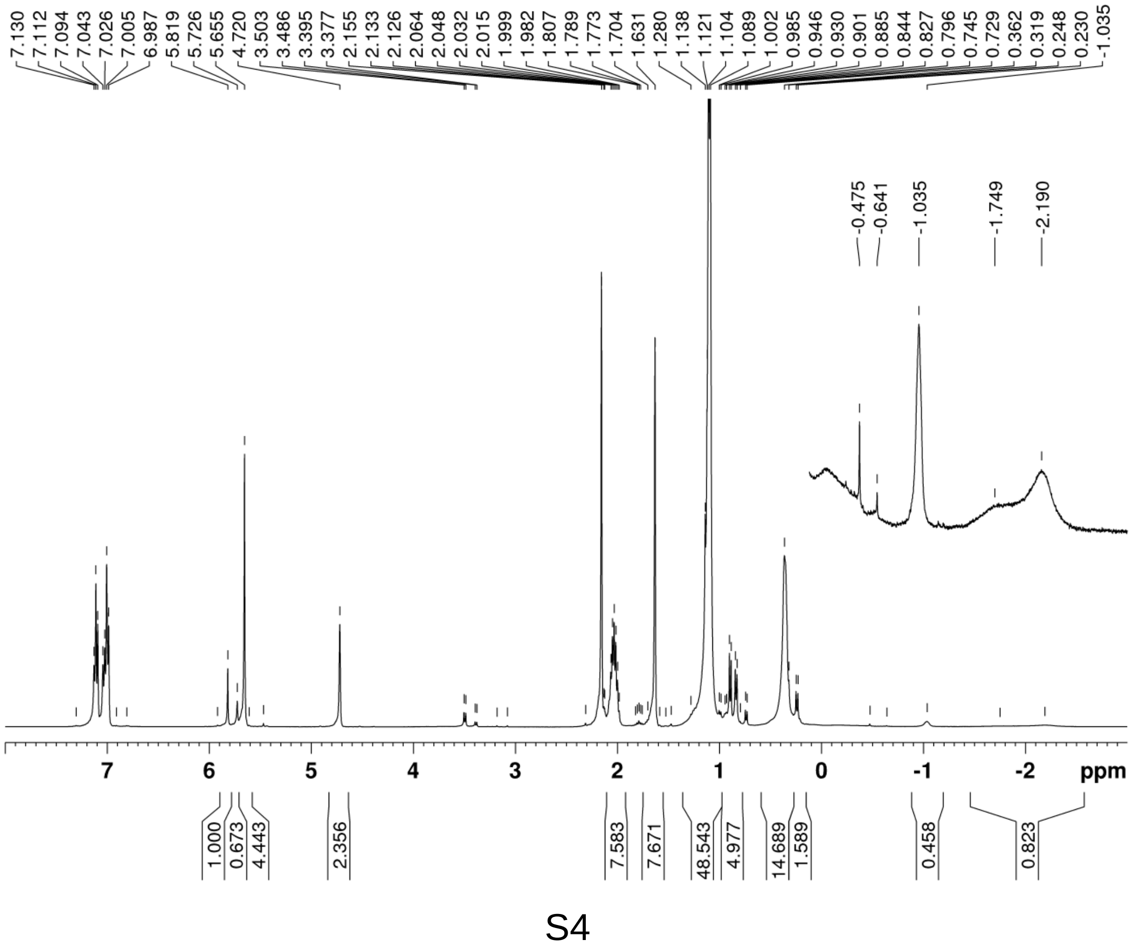

Figure S4. ^{1}H NMR of system Cp_2ZrCl_2 − $AlBu^{i}_{3}$ – MMAO-12 (1:5:12) in C_7D_8 (298 K).

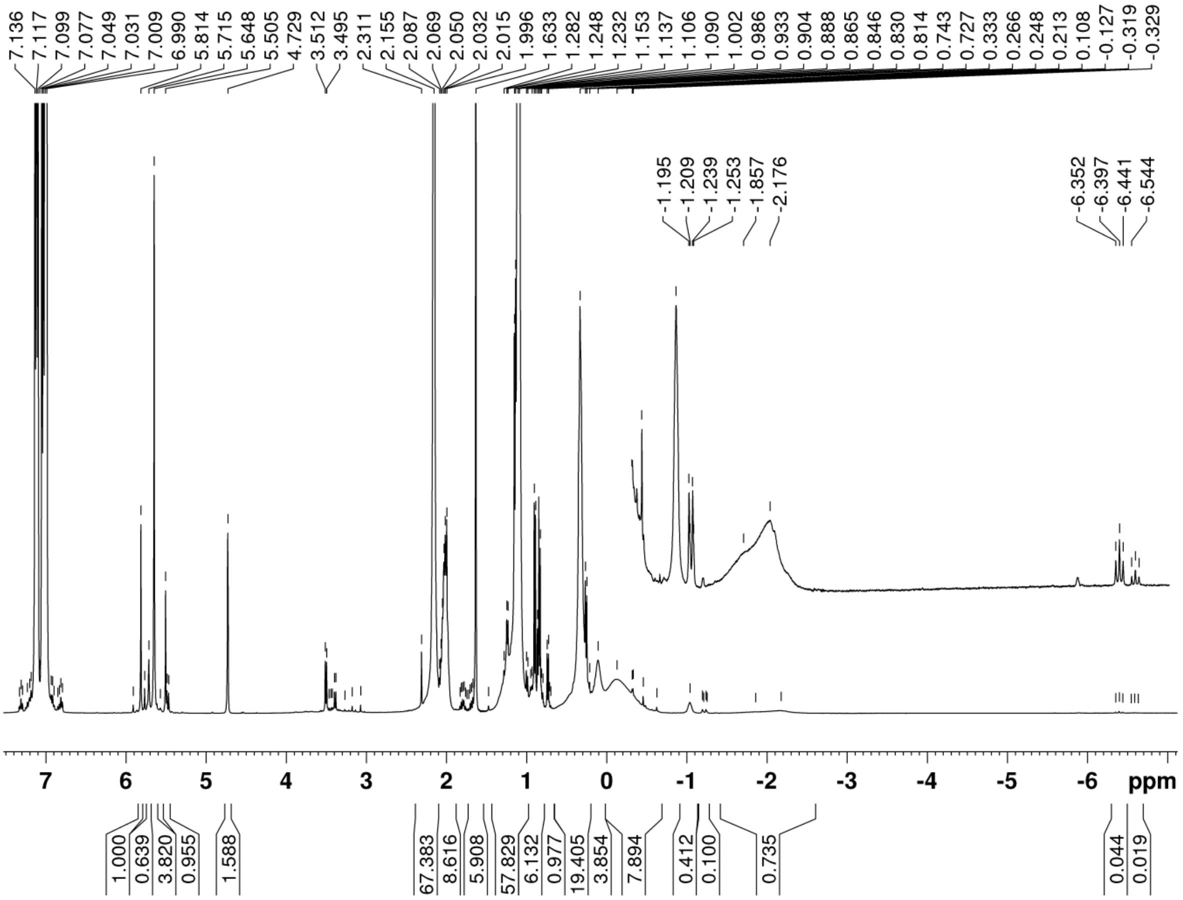

Figure S5. COSY HH of system Cp_2ZrCl_2 − $AlBu^{i}_{3}$ – MMAO-12 (1:5:12) in C_7D_8 (298 K).

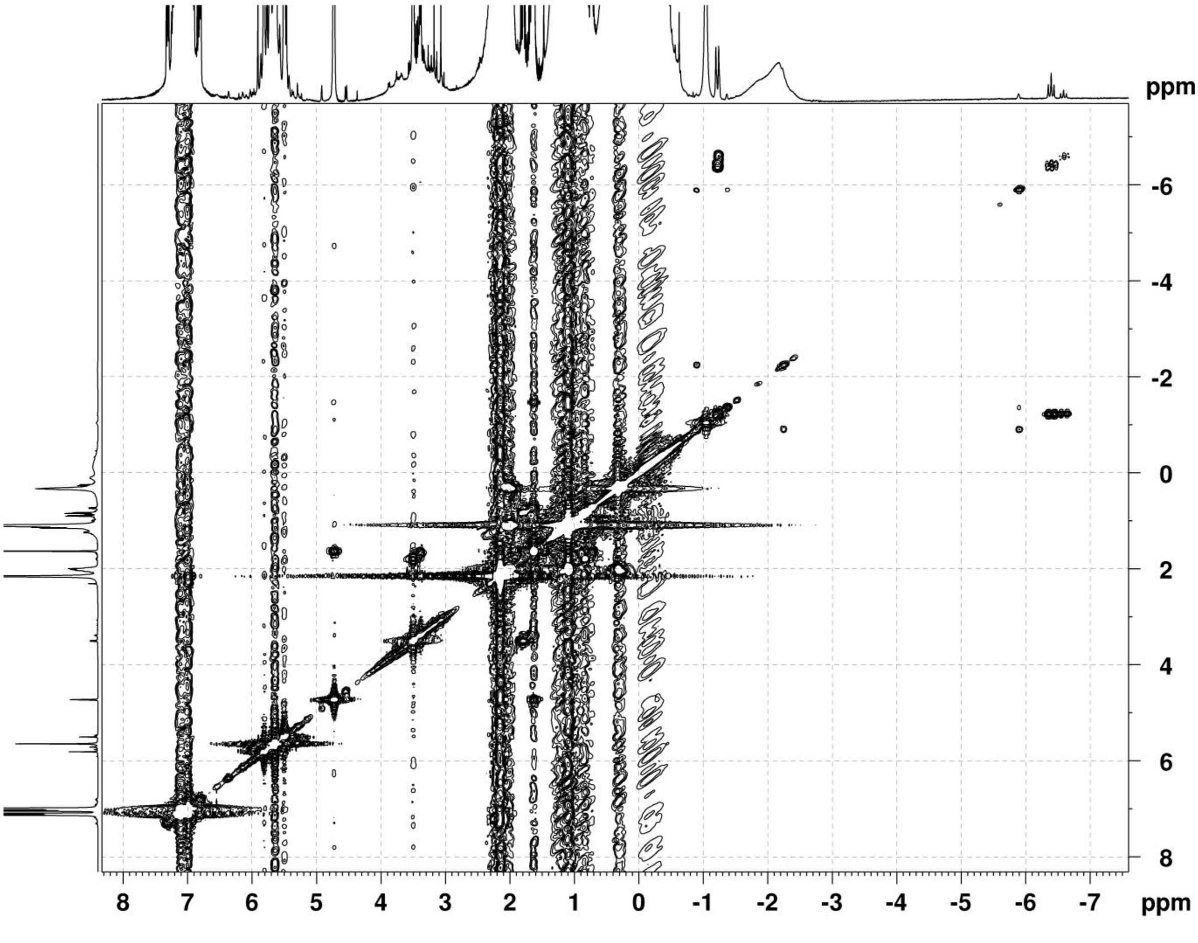

Figure S6. NOESY of system Cp_2ZrCl_2 – $AlBu^i_3$ – MMAO-12 (1:5:12) in C_7D_8 (298 K).

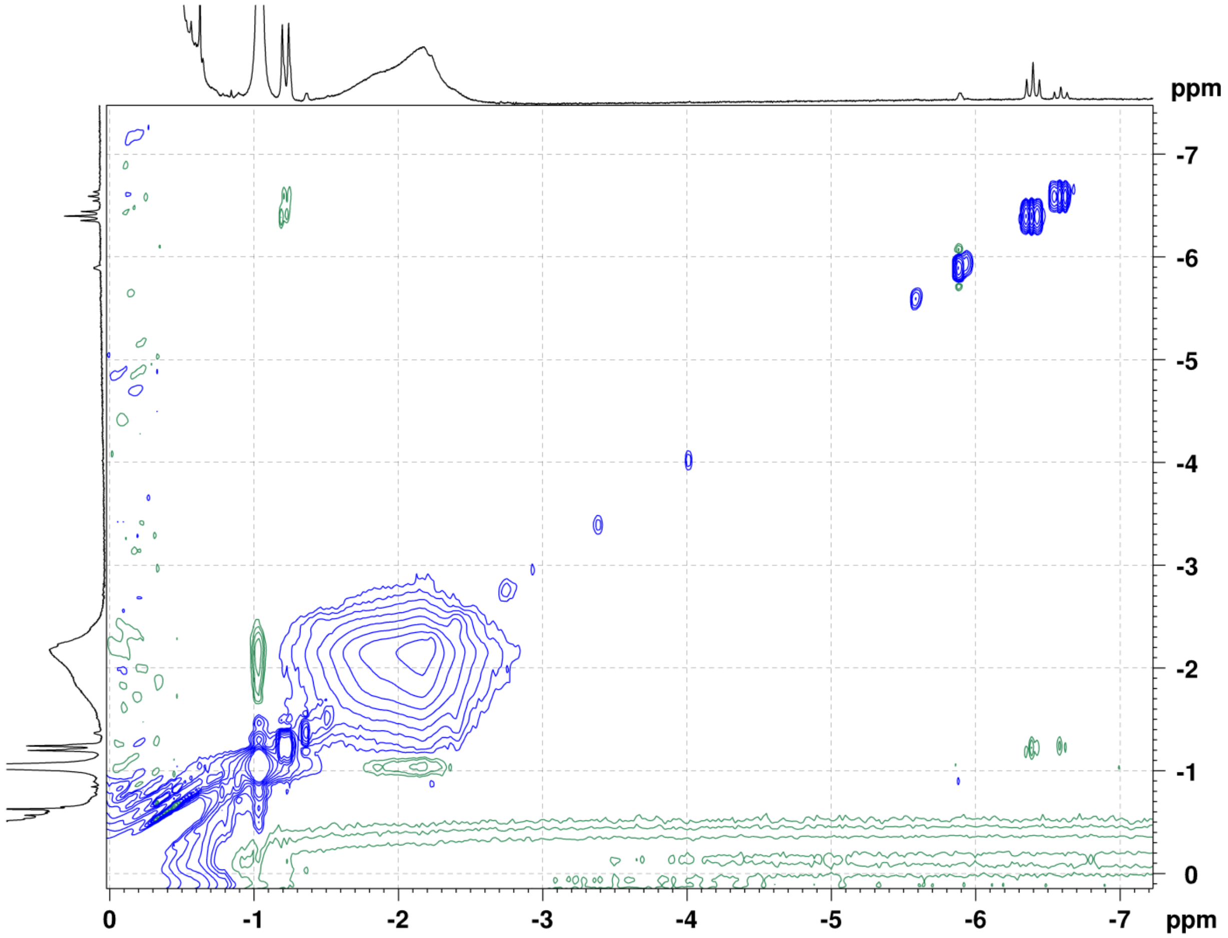

Figure S7. ^1H NMR of system Cp_2ZrCl_2–$HAlBu^i_2$–MMAO-12 in C_7D_8 (T= 298 K): a) [Zr]:[Al]:[Al_{MAO}]= 1:1.5:0; b) [Zr]:[Al]:[Al_{MAO}]= 1:1.5:1.5; c) [Zr]:[Al]:[Al_{MAO}]= 1:1.5:3.

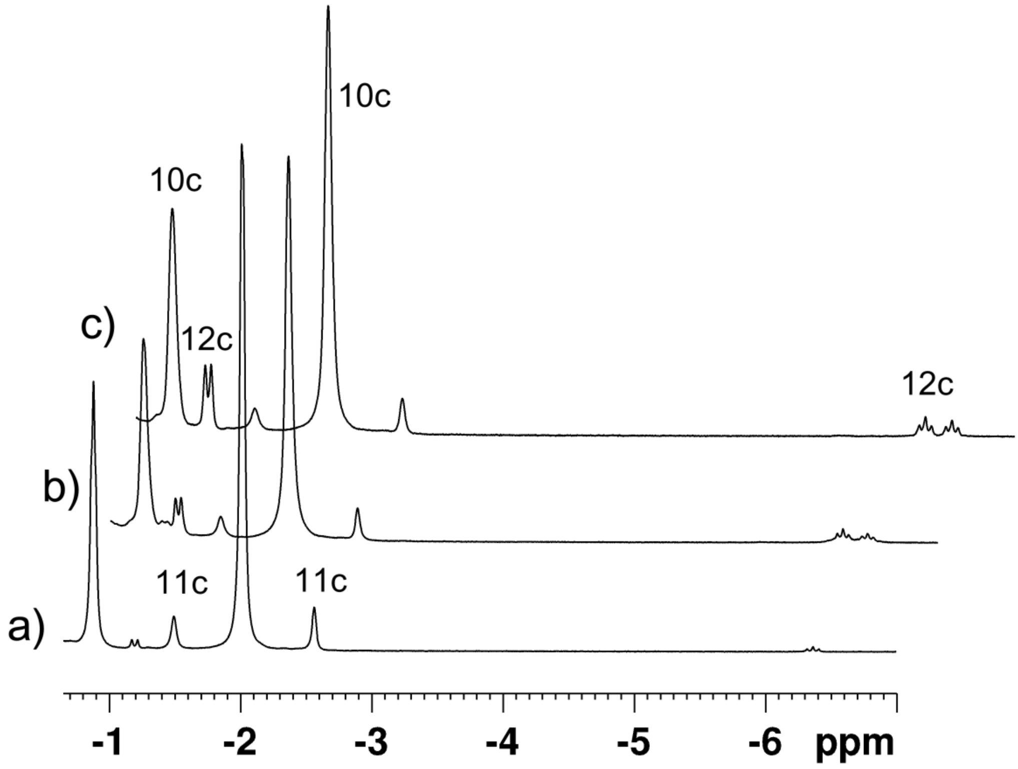

Figure S8. ^{1}H NMR of system Cp_2ZrCl_2 − $HAlBu^{i}_2$ (1:2) in C_7D_8.

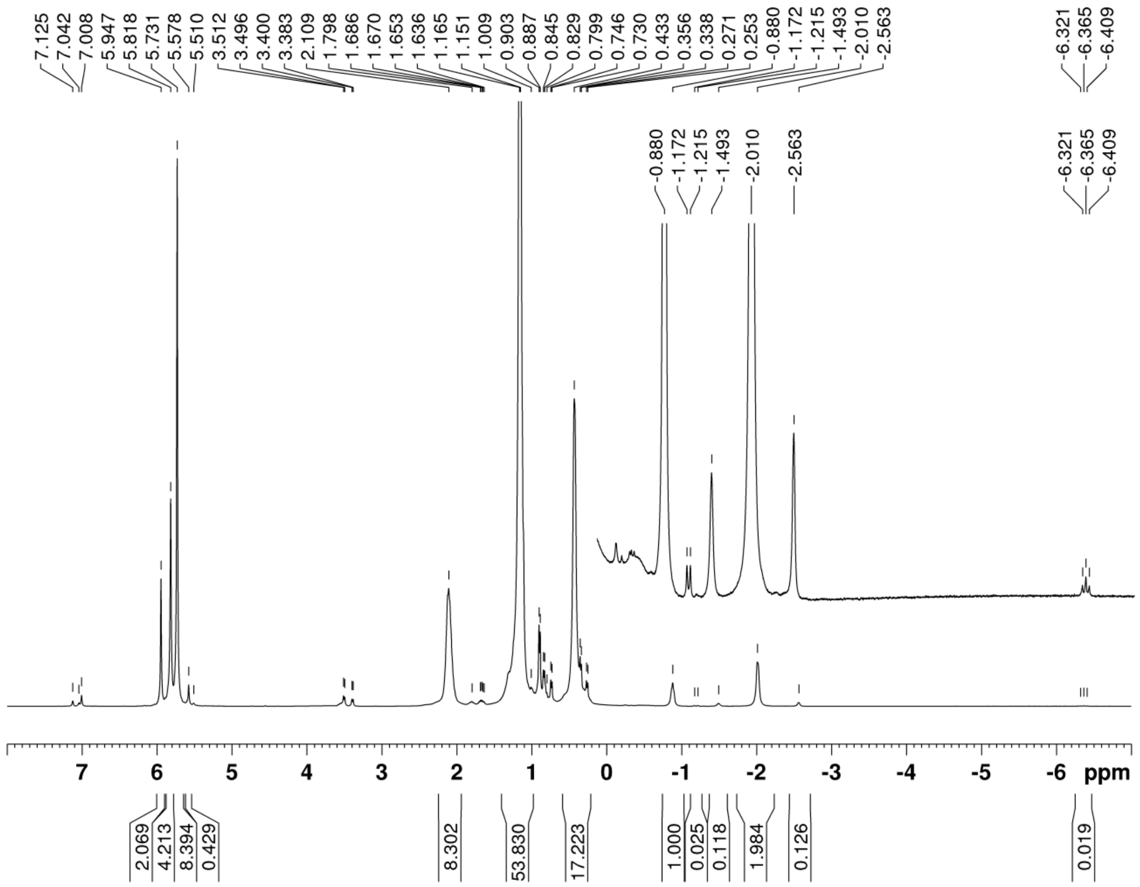

Figure S9. ^{1}H NMR of system Cp_2ZrCl_2 − $HAlBu^{i}_2$ – MMAO-12 (1:1.5:3) in C_7D_8.

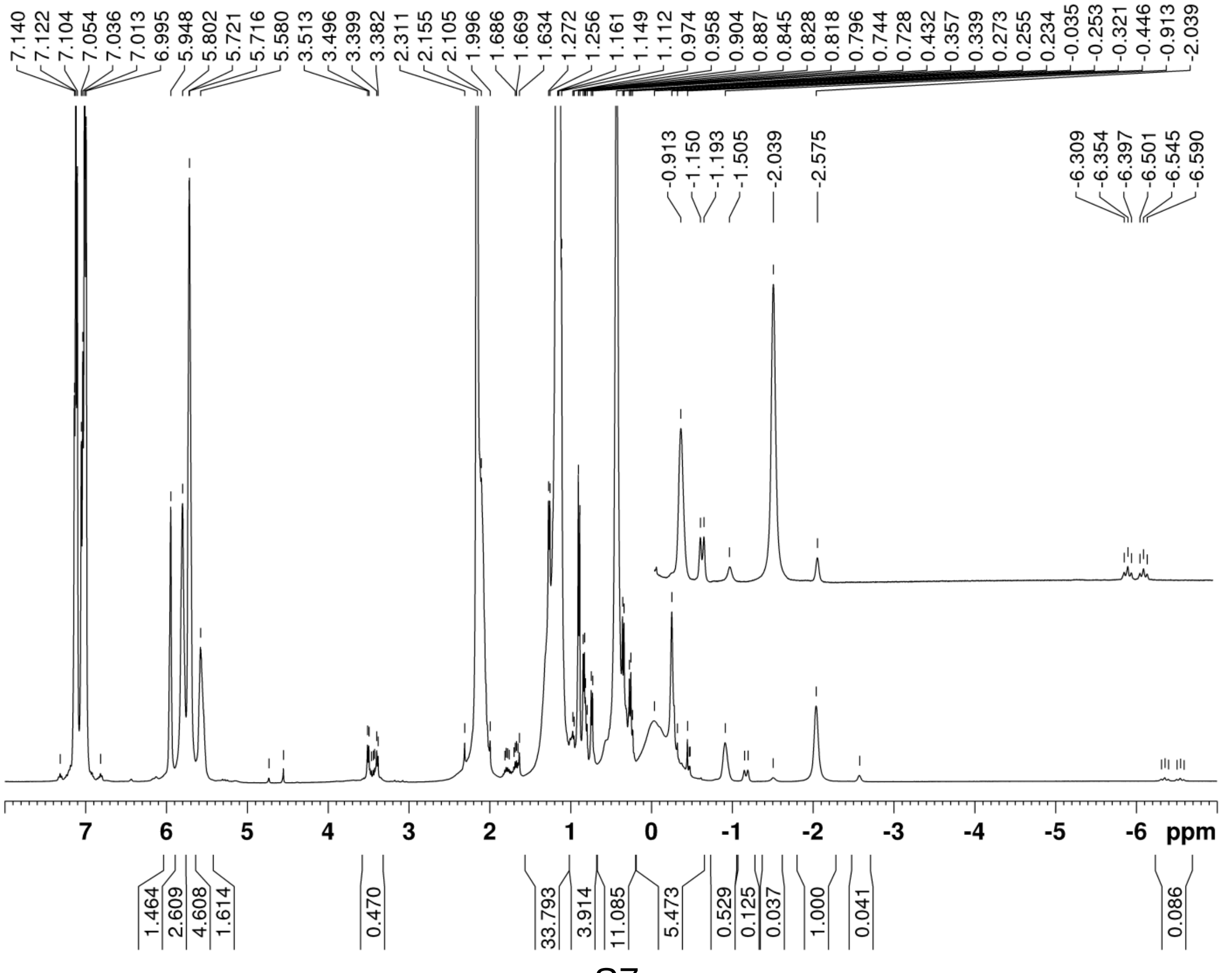

Figure S10. ^{1}H NMR of system $[Cp_2ZrH_2]_2 - ClAlMe_2$ (1:3) in C_7D_8.

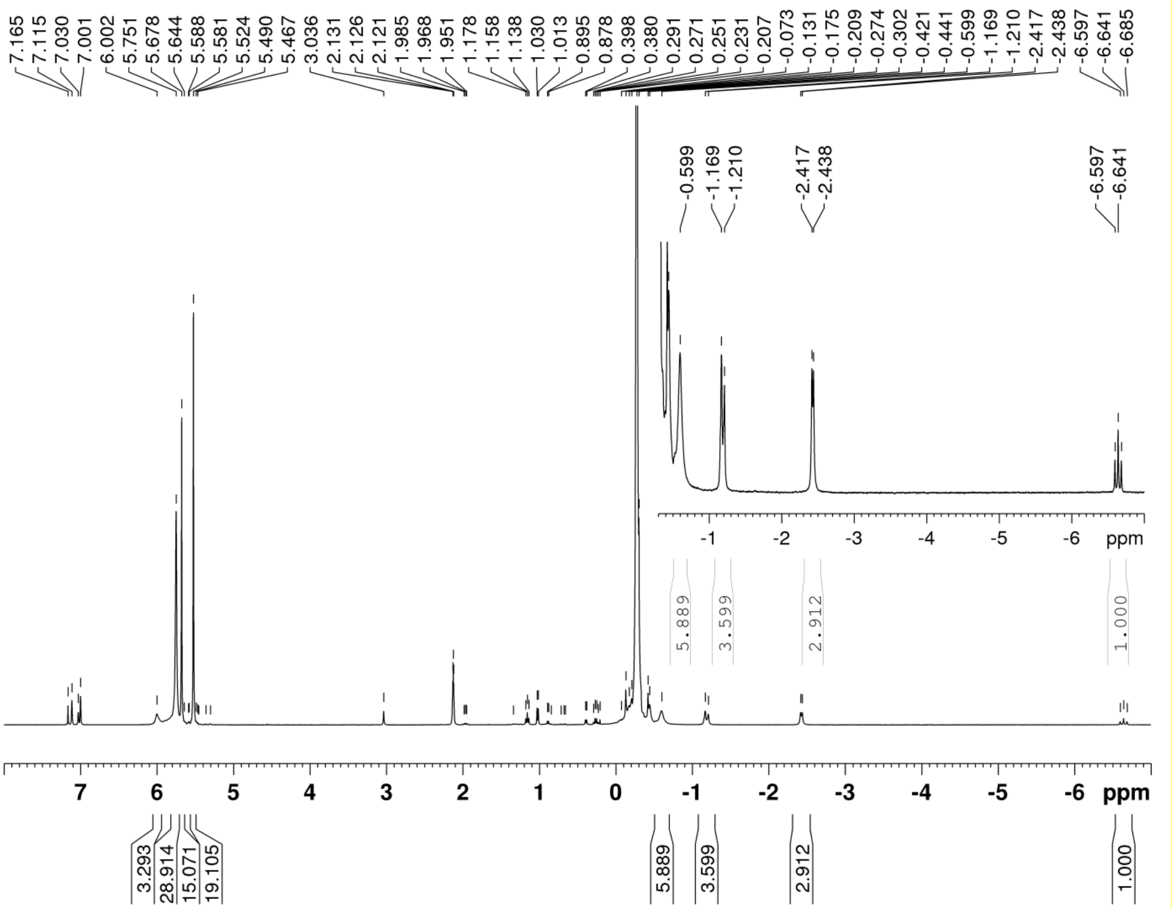

Figure S11. COSY HH of system $[Cp_2ZrH_2]_2 - ClAlMe_2$ (1:3) in C_7D_8.

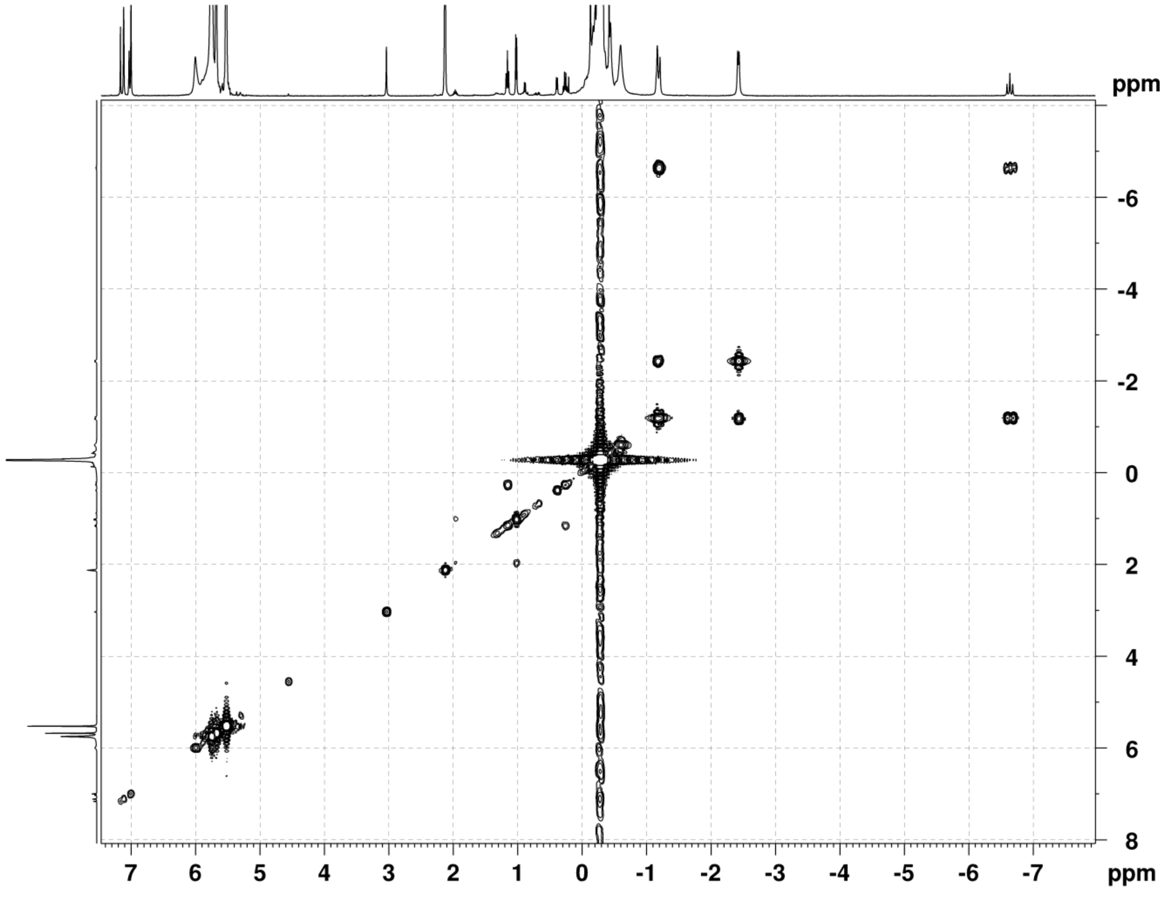

Figure S12. ^{13}C NMR of system $[Cp_2ZrH_2]_2$ − $ClAlMe_2$ (1:3) in C_7D_8.

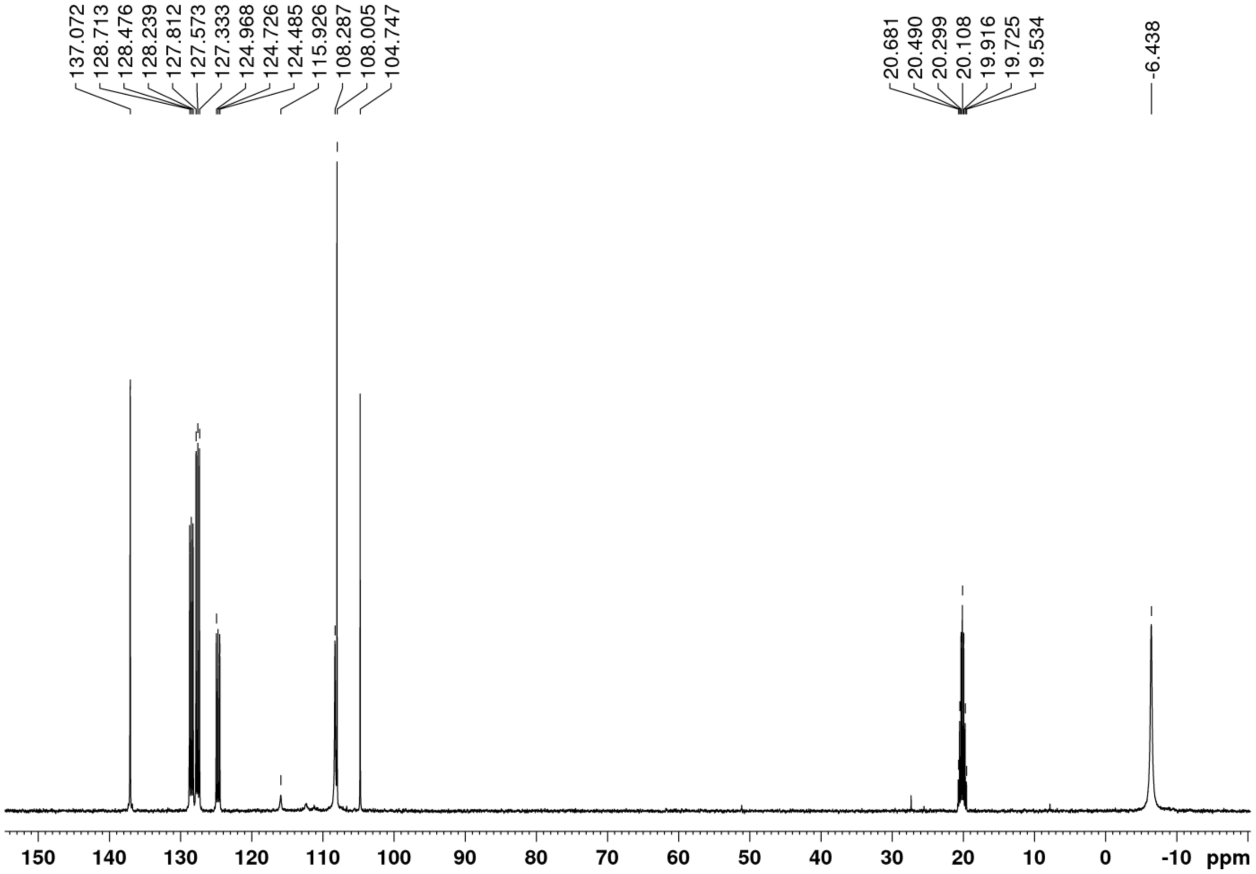

Figure S13. HSQC of system $[Cp_2ZrH_2]_2$ − $ClAlMe_2$ (1:3) in C_7D_8.

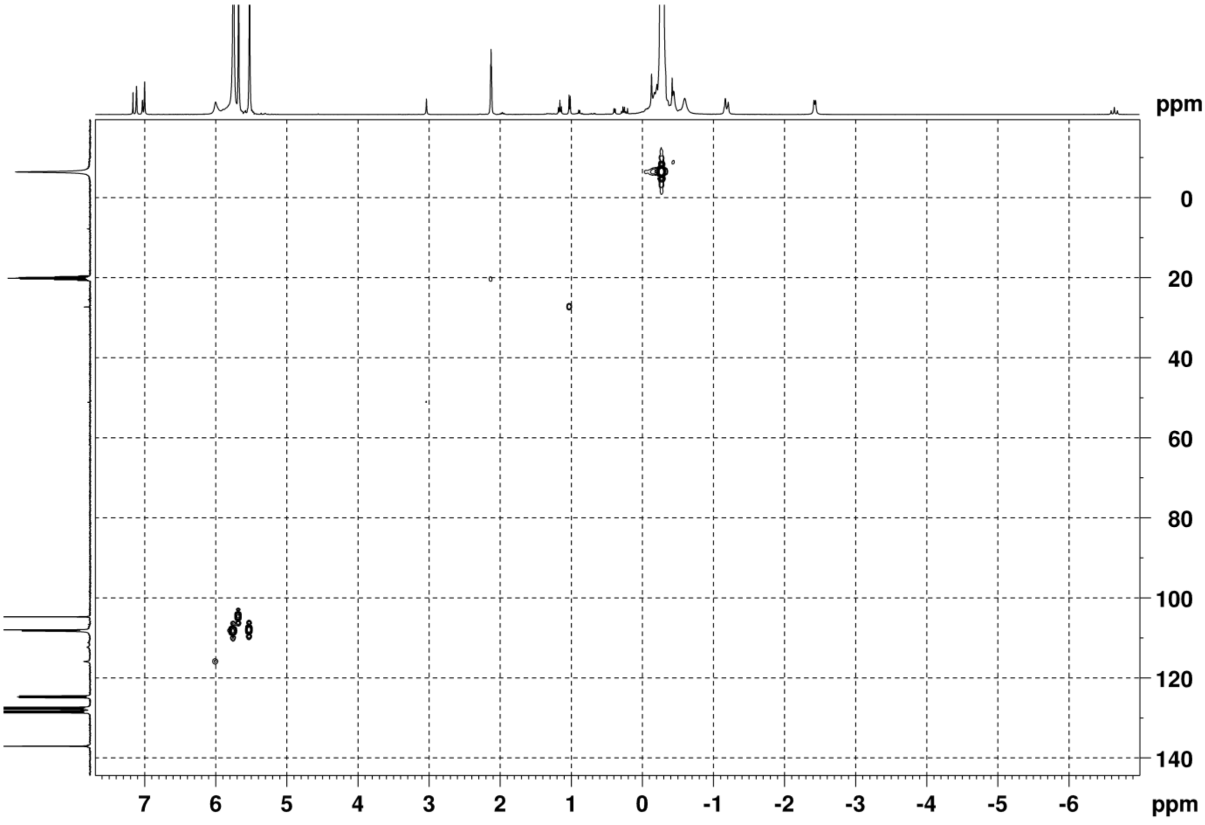

Figure S14. ^{1}H NMR of system $[Cp_2ZrH_2]_2$ − $ClAlMe_2$ – MMAO-12 (1:3:6) in C_7D_8.

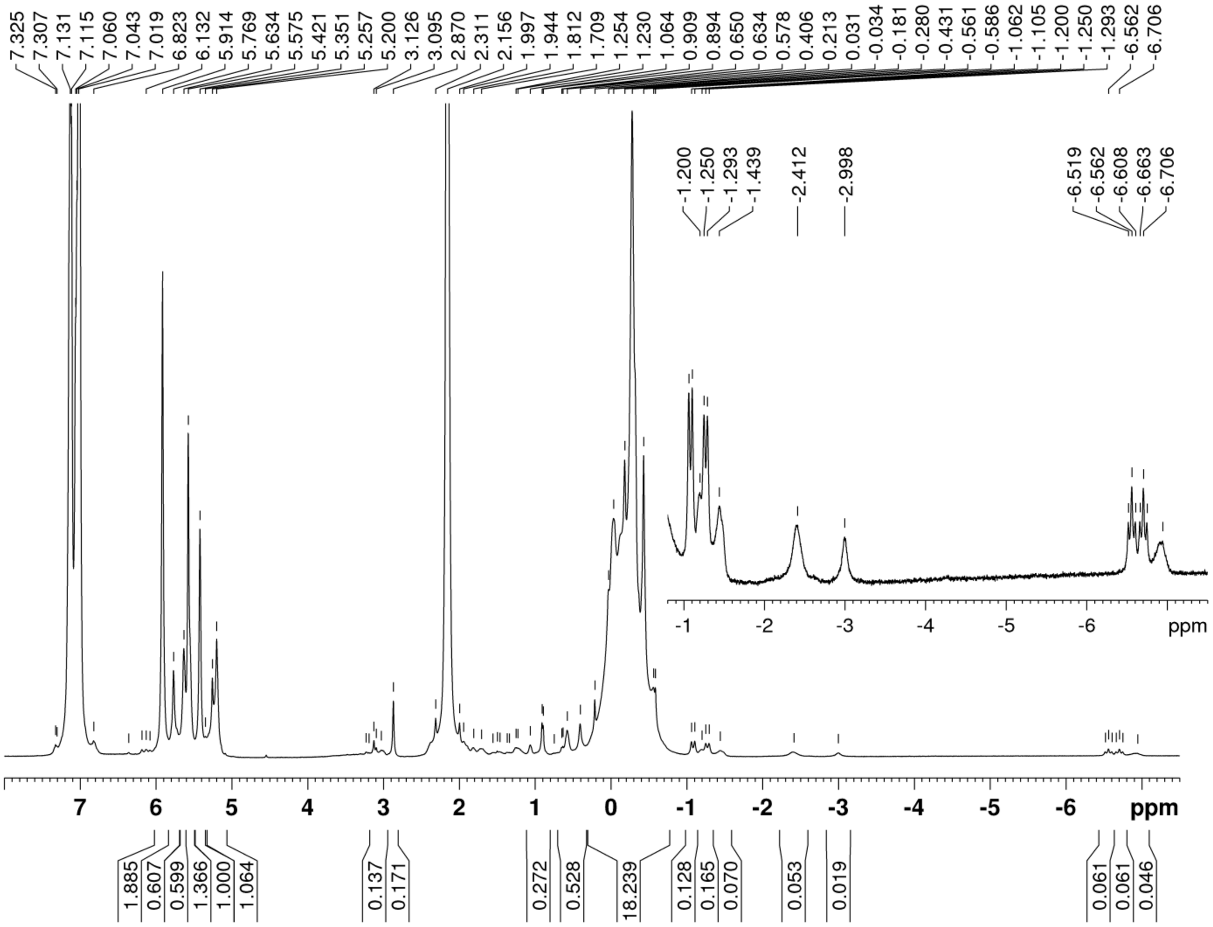

Figure S15. DOSY of system $[Cp_2ZrH_2]_2$−$ClAlMe_2$–MMAO-12 (1:3:6) in C_7D_8 (T=299.3 K).

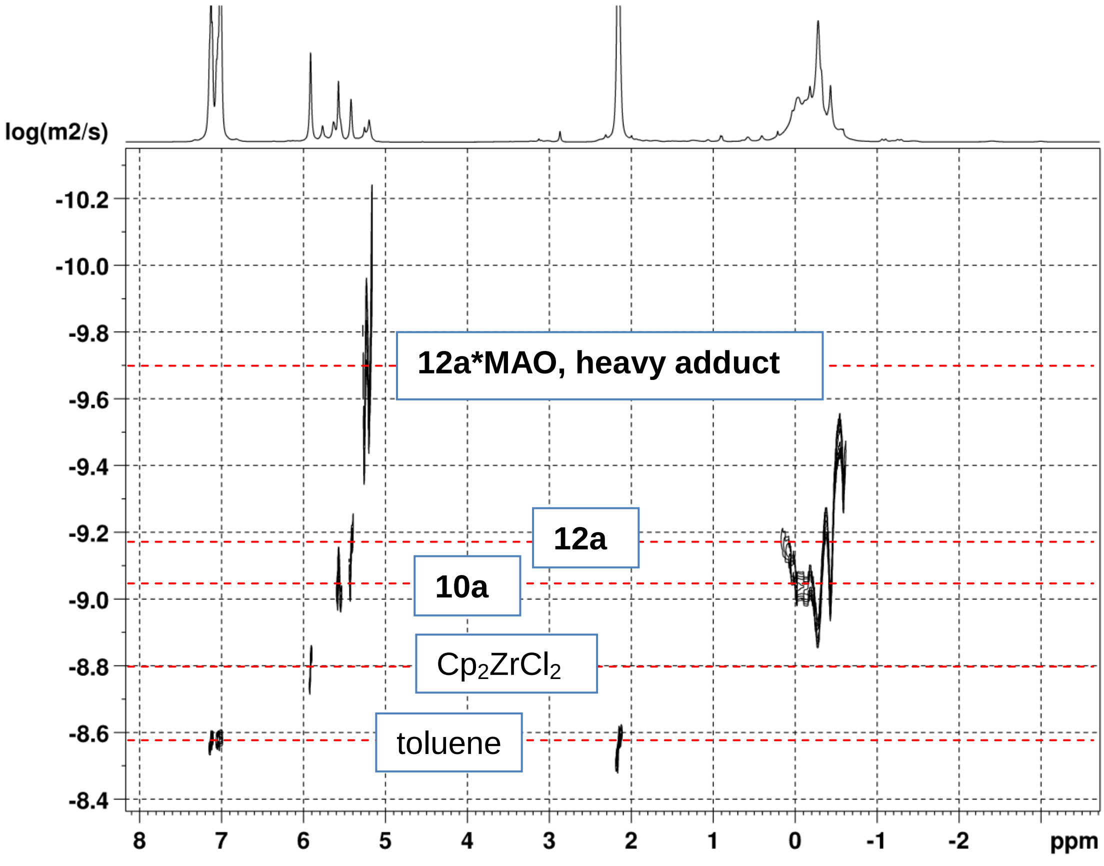

Figure S16. COSY of system $[Cp_2ZrH_2]_2$−$ClAlMe_2$–MMAO-12 (1:3:6) in C_7D_8.

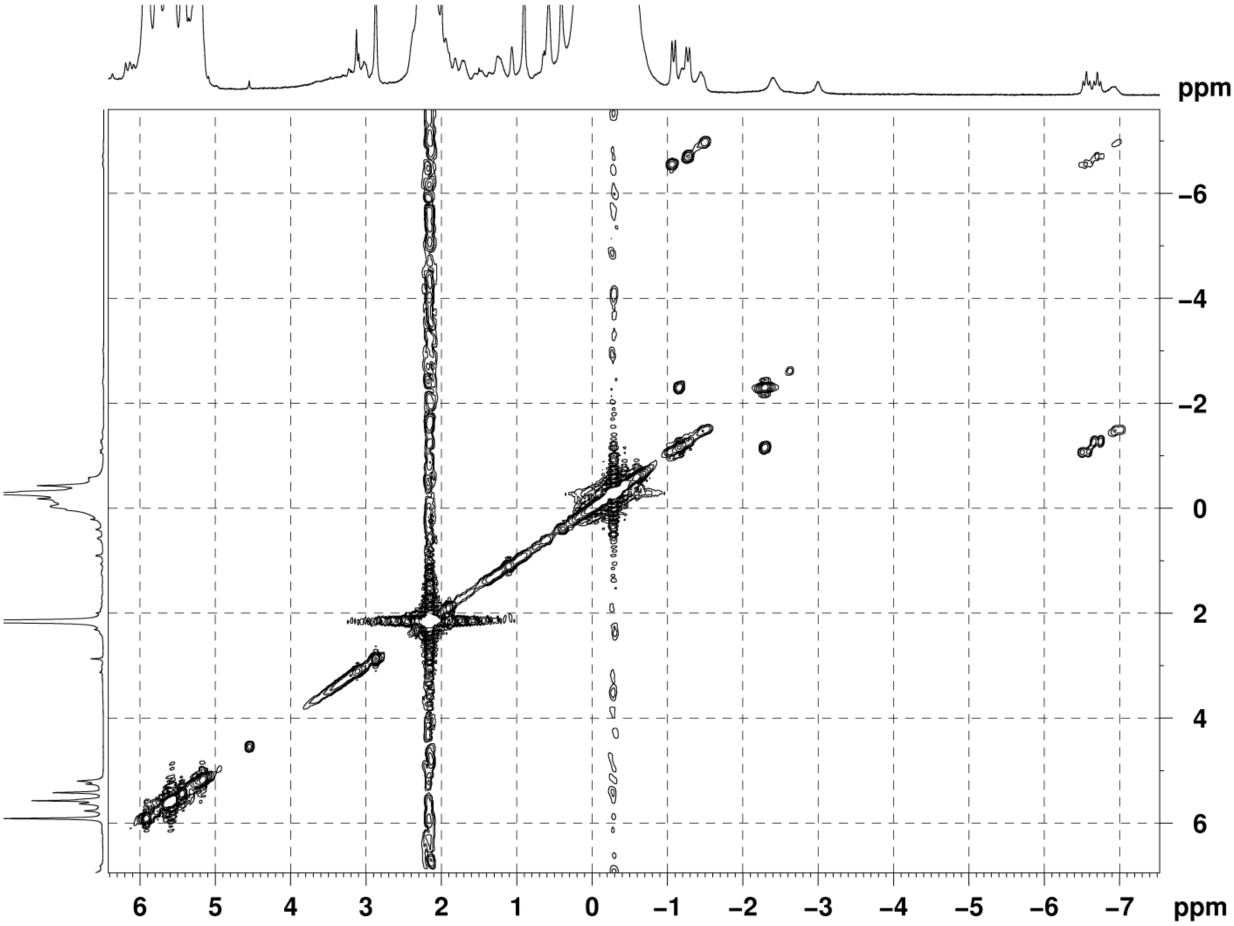

Figure S17. NOESY of system $[Cp_2ZrH_2]_2$−$ClAlMe_2$–MMAO-12 (1:3:6) in C_7D_8.

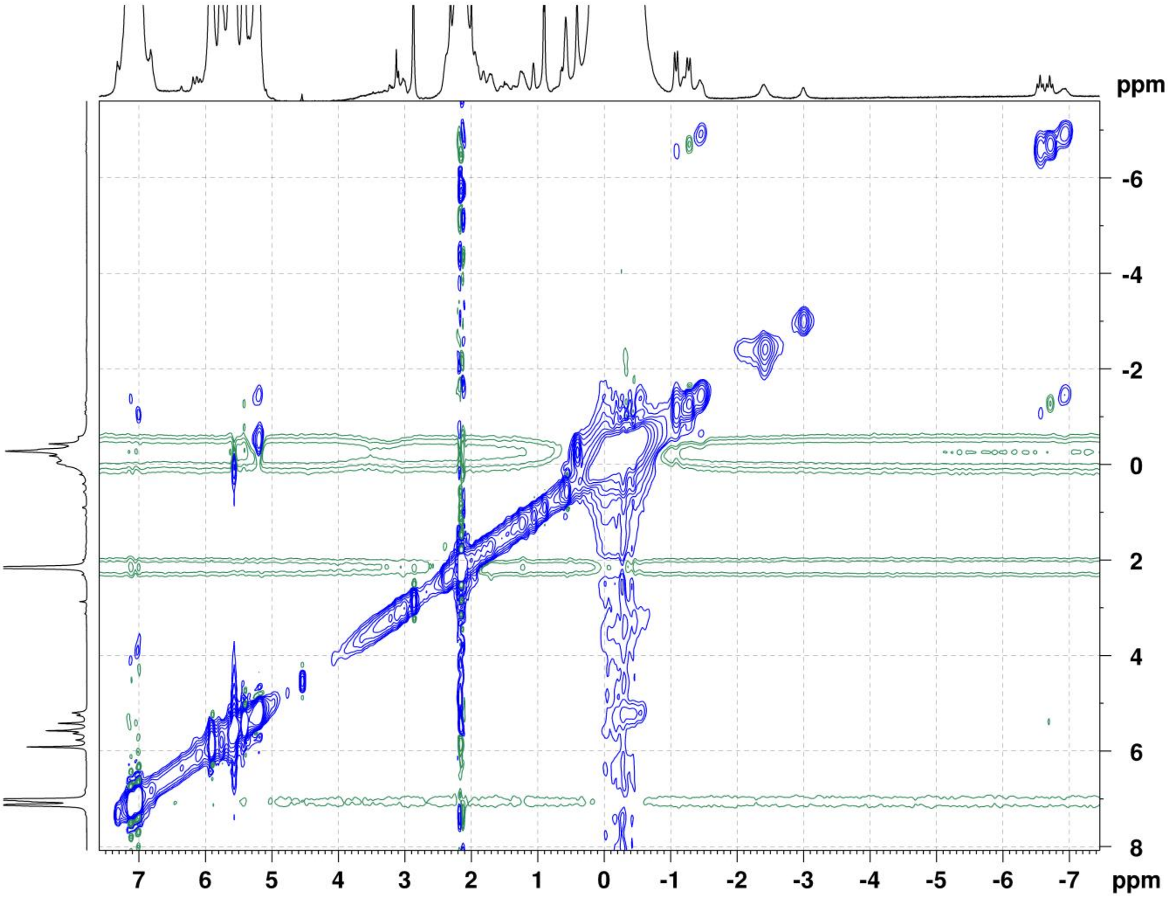

Figure S18. ^{1}H NMR of system $[Cp_2ZrH_2]_2$ − $ClAlEt_2$ (1:3) in C_7D_8.

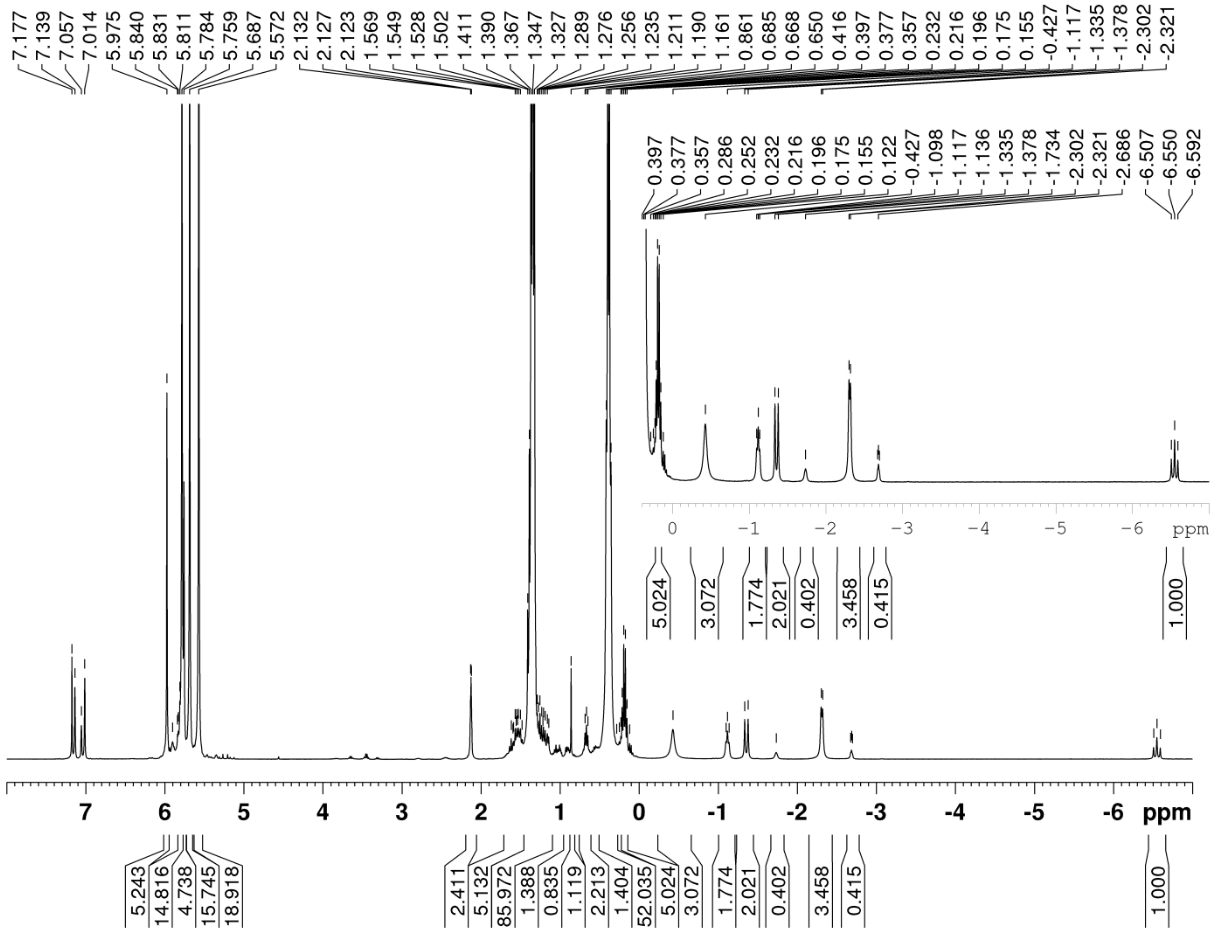

Figure S19. NMR monitoring of system $[Cp_2ZrH_2]_2$− $ClAlBu^i_2$ – 1-hexene (1:2.6:(0.7-2.4)) in C_7D_8, intensity of upfield signals is increased.

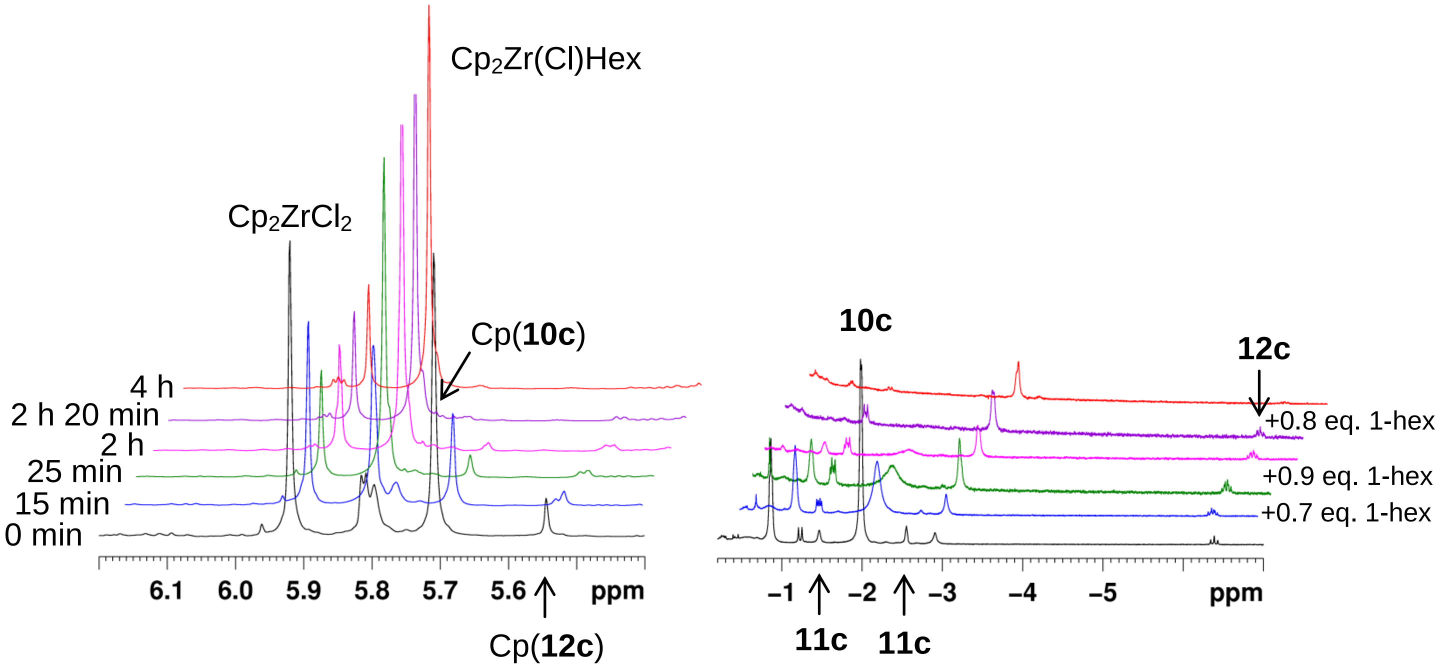

Figure S20. ^{13}C NMR of system $[Cp_2ZrH_2]_2$ − $ClAlBu^i_2$ – 1-hexene (1:2.6:(0.7-2.4)) in C_7D_8 (end of reaction).

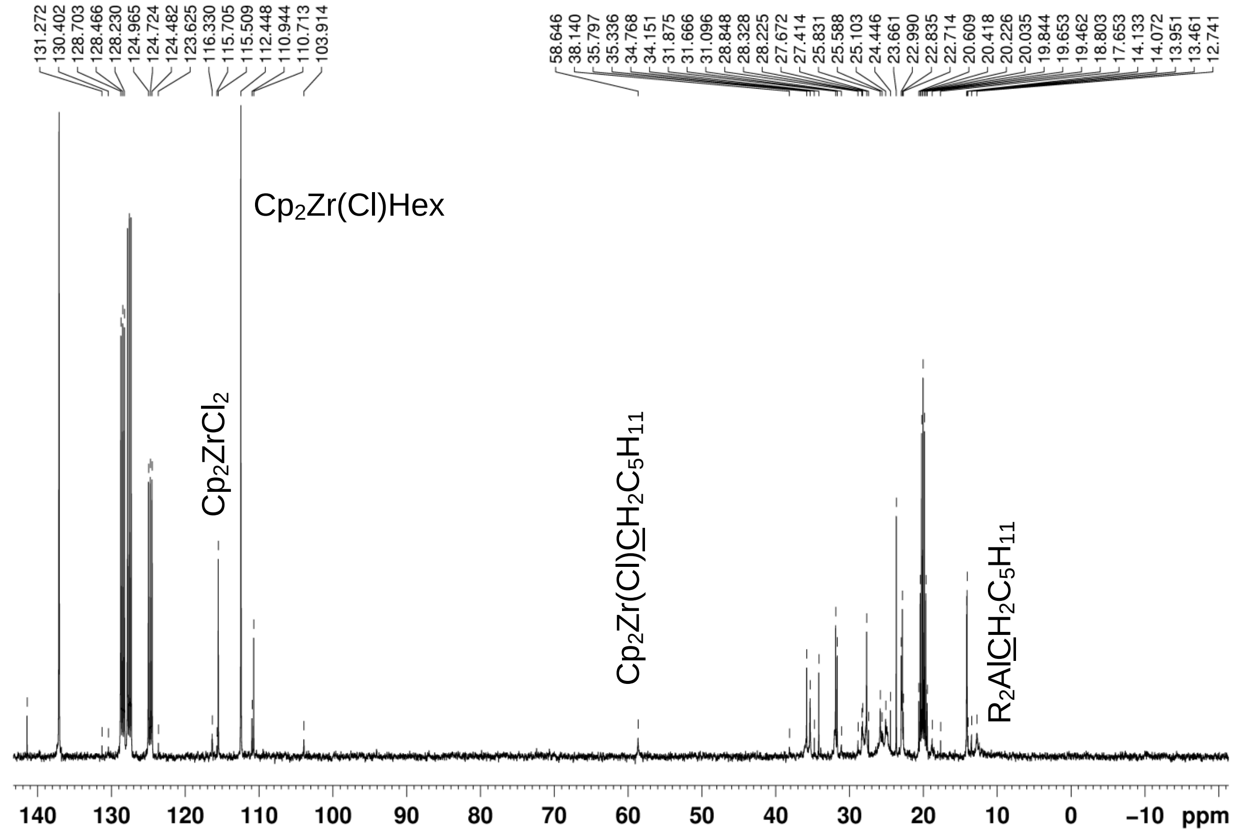

Figure S21. ^{13}C NMR of system $[Cp_2ZrH_2]_2$ − $ClAlMe_2$ – MMAO-12 – 1-hexene in C_7D_8 (end of reaction).

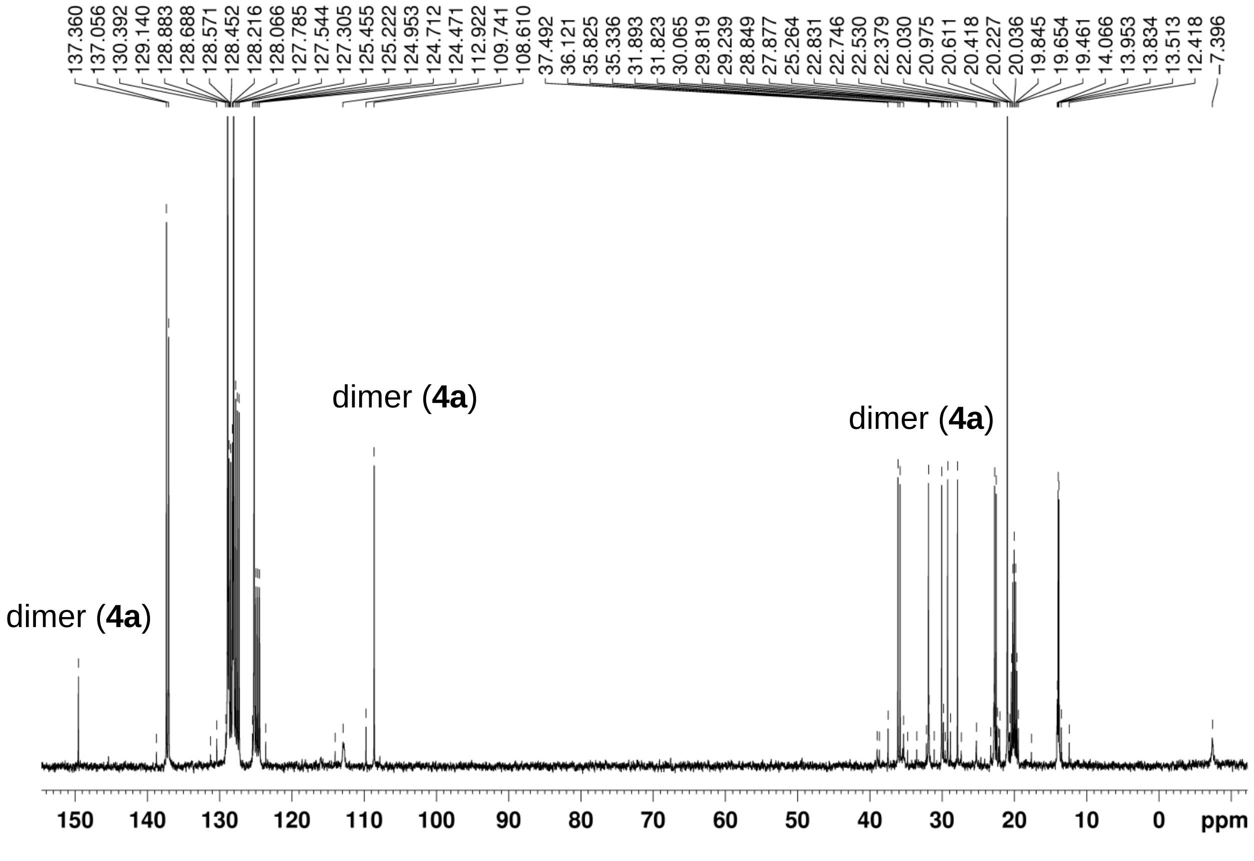

GC-MS analysis of products

Before each series of mass spectral analysis, calibration was performed using alkene-dimer mixtures with various molar concentrations to determine response factors (RF). Response factors of dimers were calculated as RF(dimer)= Slope(1-alkene)/Slope(dimer), where Slope(1-alkene) was found from the dependence Peak area (1-alkene) – Concentration (1-alkene), and Slope(dimer) from the dependence Peak area (dimer) – Concentration (dimer). 1-Alkenes were used as a standards with RF=1. Response factors of low molecular weight products **2-D**, **5-D** and **6** were taken as 1 as well. Thus, product yields were determined via peak areas multiplied by response factors. RFs of trimers were taken as RFs of dimers.

Figure S22. Example of GC-MS of products obtained in the system Cp_2ZrCl_2 – $AlMe_3$ – MMAO-12– 1-hexene (Table 1, entry 18)

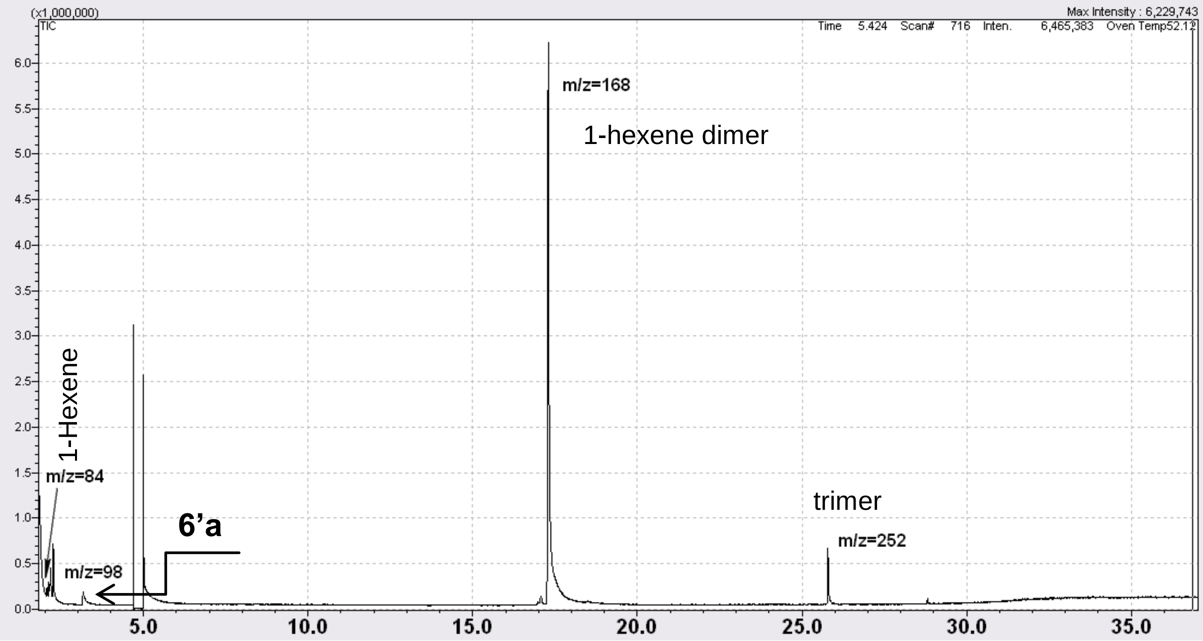

Supplement: Supplementary file 1 [file molecules-25-02216-s001.pdf]
